# Compensatory enhancement of input maintains aversive dopaminergic reinforcement in hungry *Drosophila*

**DOI:** 10.1016/j.neuron.2024.04.035

**Published:** 2024-05-24

**Authors:** Eleonora Meschi, Lucille Duquenoy, Nils Otto, Georgia Dempsey, Scott Waddell

**Affiliations:** 1 University of Oxford, Centre for Neural Circuits and Behaviour, Tinsley Building, Mansfield Road, Oxford OX1 3SR, UK

## Abstract

Hungry animals need compensatory mechanisms to maintain flexible brain function, while modulation recon-figures circuits to prioritize resource seeking. In *Drosophila*, hunger inhibits aversively reinforcing dopaminergic neurons (DANs) to permit the expression of food-seeking memories. Multitasking the reinforcement system for motivation potentially undermines aversive learning. We find that chronic hunger mildly enhances aversive learning and that satiated-baseline and hunger-enhanced learning require endocrine adipokinetic hormone (AKH) signaling. Circulating AKH influences aversive learning via its receptor in four neurons in the ventral brain, two of which are octopaminergic. Connectomics revealed AKH receptor-expressing neurons to be upstream of several classes of ascending neurons, many of which are presynaptic to aversively reinforcing DANs. Octopaminergic modulation of and output from at least one of these ascending pathways is required for shock- and bitter-taste-reinforced aversive learning. We propose that coordinated enhancement of input compensates for hunger-directed inhibition of aversive DANs to preserve reinforcement when required.

## Introduction

Internal states such as those manifesting during periods of hunger, thirst, aggression, and sleep are known to rely on the concerted actions of peptide and amine neuromodulators/neurohormones throughout the body. In the brain, these molecules can radically change the dynamics and routing of activity by altering the excitability and functional connectivity of neurons within their larger networks. Sometimes modulators exert brain-wide effects by simultaneously signaling through receptor(s) expressed at multiple locations in the brain, whereas others appear to function more locally.^1,2^ It is therefore important to understand how modulatory systems work together to facilitate the dominant behavioral state. Moreover, it is unclear how state-dependent modulation of different neurons within a circuit is co-ordinated to maintain the animal’s flexible use of the same circuits for alternative purposes.

In *Drosophila*, several conserved neurohormones/modulators have been identified that play, sometimes antagonistic, roles in the physiological and behavioral regulation of energy homeostasis.^2,3^ The *Drosophila* insulin-like peptides (dILPs) and adipokinetic hormone (AKH) are functionally analogous to insulin and glucagon in mammals, which are central to glucose and lipid metabolism.^4,5^ Four dILPs are released from neurosecretory insulin-producing cells in the medial protocerebrum, and the single insulin receptor (InR) is very broadly expressed throughout the brain.^6,7^ By contrast, AKH is produced outside the brain by a group of endocrine cells in the corpora cardiaca (CC) that are associated with the esophagus and considered to be the functional orthologs of mammalian-pancreatic α cells.^5,8^ Unlike the InR, neuronal expression of the AKH receptor (AKHR) appears restricted to only four neurons in the subesophageal zone (SEZ) of the ventral brain and to sweet-sensing gustatory neurons.^9,10^ To date octopamine (OA) release from AKHR neurons has been implicated in starvation-induced behavioral hyperactivity.^10^ However, it remains unclear whether and how these apparently local AKHR neurons affect other brain functions.

Neuropeptide F (dNPF), the fly equivalent of mammalian neuropeptide Y, and leukokinin have also been implicated in energy homeostasis.^11,12^ In addition, their release from neurons located in the dorsal protocerebrum contributes to hunger-state-dependent control of food-seeking memories.^13,14^ In both cases, a critical site of action of these neuropeptides is inhibitory modulation of different subsets of aversively reinforcing dopaminergic neurons (DANs), which innervate discrete compartments of the fly’s mushroom bodies (MBs). Modulating the dopaminergic reinforcement system in this way potentially undermines its ability to function in aversive learning. Study of motivational dopaminergic control systems therefore provides an opportunity to understand how neuronal circuit function remains flexible when network properties are altered by changing internal states.

Here, we demonstrate a role for endocrine AKH in aversive ol-factory learning. AKH regulates memory acquisition via the activation of four AKHR-expressing neurons in the SEZ, two of which are octopaminergic. We show that these AKHR neurons are synaptically connected to multiple types of ascending neurons, some of which provide direct input to aversively reinforcing DANs. AKHR neuron-directed octopaminergic modulation of SEZ output neurons supports normal and hunger-enhanced levels of aversive learning. This study therefore reveals an unexpected role for nutrient-state-responsive AKH and AKHR signaling as critical modulators of an input pathway to aversively reinforcing DANs. We propose this mechanism maintains the ability of DANs to direct aversive learning while they are engaged for motivational purpose.

## Results

### Starvation enhances aversive olfactory learning

*Drosophila* can learn to associate odors with electric shock punishment or sugar (or other nutrient) reward.^15–17^ After learning, flies preferentially avoid or approach the previously reinforced odor. The internal state of hunger is critical for formation and expression of olfactory sugar-seeking memory.^13,16,18^ Although expression of shock-reinforced aversive memory appears to be state-independent, relative hunger state can alter aversive memory persistence.^19–21^

**Figure 1:**
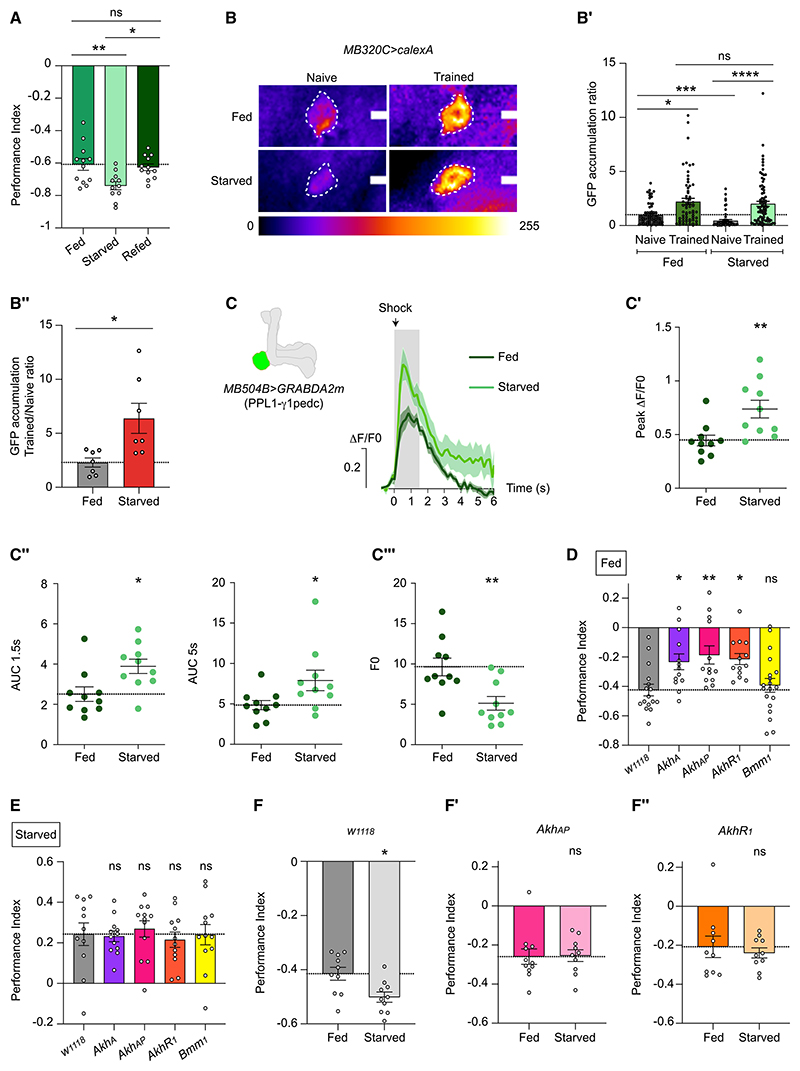
Starvation-enhanced aversive learning requires AKH signaling (A) Immediate aversive memory in fed, starved (24 h on 1% agar), and starved then refed (3 h before training) adult wild-type flies (*n* ≥ 11). (B) Examples of CaLexA recorded PPL1-γ1pedc activity before and after aversive training in fed and starved flies. UAS-*CaLexA* driven with MB320C split-GAL4. Scale bars, 10 μm. (B′) Quantification of CaLexA recorded activity, *n* ≥ 61. Each data point represents value from a single PPL1-γ1pedc DAN. (B″) CaLexA GFP accumulation ratio of aversively trained versus naive flies, fed and starved, *n* = 7. Each data point represents mean value from independent experiments. (C) Mean shock-evoked (90 V) ΔF/F_0_ dopamine transients measured *in vivo* from PPL1-γ1pedc DANs of fed and starved flies, *n* = 10. UAS-*GRAB*_*DA2m*_ driven by MB504B-split-GAL4. Gray rectangle marks 1.5 s shock. (C′) Quantification of peak ΔF/F_0_ dopamine transients in (C). (C″) Quantification of mean area under the curve during 1.5 s shock and 5 s after onset of shock from recordings in (C). (C‴) Quantification of baseline GRAB_DA2m_ values, F_0_, in fed and starved flies in (C). (D) Immediate aversive memory of fed *w*^*1118*^, *Akh^A^*, *Akh^AP^*, *AkhR^1^*, and *Bmm^1^* flies, *n* ≥ 13. (E) Immediate appetitive memory of odor-sugar-trained *w*^*1118*^, *Akh^A^*, *Akh^AP^*, *AkhR^1^*, and *Bmm^1^* flies, *n* ≥ 11. (F) Immediate aversive memory is enhanced by starvation in wild-type but not (F′) *Akh^AP^* or (F″) *AkhR^1^* mutants, *n* ≥ 10. Data presented as mean ± standard error of mean (SEM). Individual data points of behavioral graphs represent independent groups of approximately 200 flies. Asterisks denote significant differences: *p < 0.05, **p < 0.01, ***p < 0.001, ****p < 0.0001. See also Figure S1.

To investigate whether starvation modulates aversive olfactory learning, we trained and tested wild-type flies subjected to 24 h food deprivation. Unexpectedly, chronically starved flies exhibited mildly enhanced aversive olfactory memory compared with *ad libitum*-fed (satiated) flies when tested immediately after training (Figure 1A). Odor and shock acuity were not altered in hungry naive flies (Figures S1A–S1C), suggesting selectivity to aversive olfactory learning. Moreover, learning scores returned to that of satiated flies if starved flies were refed for 3 h before training (Figure 1A), indicating that hunger acutely elevates aversive learning. In addition, water deprivation did not enhance aversive learning over that of food-sated flies, demonstrating that enhanced learning is not a general response of deprivation (Figure S1D).

PPL1-γ1pedc (PPL101) DANs are required to relay the reinforcing effects of electric shock, and their activity is hunger modulated.^13,20,22–24^ We therefore compared PPL1-γ1pedc neuronal activity before and after aversive training in fed and starved flies using CaLexA,^25^ which reflects a cumulative record of neuronal activation (Figure 1B). Since CaLexA is a transcriptional reporter of intracellular Ca^2+^, we processed brains 3 h after training to allow GFP transcription and translation (starvation-induced memory enhancement remains at least 3 h after training [Figure S1E], but not 24 h later [Figure S1F], consistent with Hirano et al.^19^). Naive starved flies exhibited reduced baseline PPL1-γ1pedc CaLexA signal to that of naive fed flies, consistent with hunger-dependent inhibition.^13,20^ By contrast, aversive learning produced similar elevated Ca-LexA signal in PPL1-γ1pedc DANs in fed and starved flies (Figures 1B and 1B′), which translates to a greater increase from baseline after training in starved flies (Figure 1B″). We also acutely measured baseline and shock-evoked dopamine (DA) release from PPL1-γ1pedc DANs using transgenic expression of the fluorescent GRAB_DA_ sensor^26^ (Figures 1C–1C‴). These recordings again revealed lower PPL1-γ1pedc DAN baseline activity in starved over-fed flies (Figures 1C‴and S1G‴). In addition, shock evoked significantly enhanced DA release in starved versus fed flies (Figures 1C–1C″). Interestingly, fed and starved animals showed comparable odor-evoked DA release from PPL1-γ1pedc DANs (Figures S1G–S1G″), consistent with hunger specifically modulating a shock-triggered input pathway. Prior work showed PPL1-γ1pedc DAN calcium responses, the magnitude of their induced learning-relevant depression of connections between odor-activated Kenyon cells (KCs) and downstream output neurons, and aversive learning scores correlate with shock intensity.^27^ Therefore, larger shock-evoked DA signals should enhance/maintain aversive learning in food-deprived flies (Figure 1A).

### AKH signaling is required for aversive olfactory learning

AKH is released from the CC into the hemolymph to signal hunger and restore glycemia.^8^ We therefore tested aversive learning in flies harboring mutations in the AKH (*Akh^A^* and *Akh^AP^*) and AKHR (*AkhR^1^*) genes.^28,29^ The *Akh^A^* allele deletes two C-terminal amino acids of AKH peptide, whereas *Akh^AP^* is a null that removes AKH and AKH-precursor-related peptide coding regions. *AkhR^1^* is a null mutant for AKHR.

AKH pathway mutant flies displayed impaired immediate aversive memory compared with controls (Figure 1D; there was no difference between sexes, Figure S1H). Whereas null *AkhR^1^* and *Akh^AP^* mutants exhibited normal odor and shock acuity, for unknown reasons, *Akh^A^* flies responded less to odor (Figures S1I–S1K). We therefore used *Akh^AP^* and *AkhR^1^* flies for further experiments. Perhaps surprisingly, *Akh* and *AkhR* mutants displayed normal sugar-reward learning (Figure 1E). Since *Akh^AP^* and *AkhR^1^* mutants are phenotypically obese,^28,29^ with increased triglyceride (TAG) levels (Figure S1L), we tested whether defective aversive learning might result from obesity. Flies mutant for the lipase Brummer (*Bmm^1^*) exhibit comparable obesity to *AkhR^1^* mutant flies^28^ (Figure S1L), but their aversive learning and odor and shock acuity were indistinguishable from controls (Figures 1D and S1I–S1K). Hence, AKH/AKHR may promote aversive olfactory learning independently of lipolytic function.

We also tested whether AKH/AKHR were required for elevated aversive learning following starvation. Unlike control *w*^*1118*^ flies, *Akh^AP^* and *AkhR^1^* mutants showed equivalent aversive memory when starved or fed (Figures 1F–1F″), suggesting that starvation-induced enhancement of aversive learning is AKH dependent.

### AKH-producing cells are required for aversive olfactory learning

**Figure 2:**
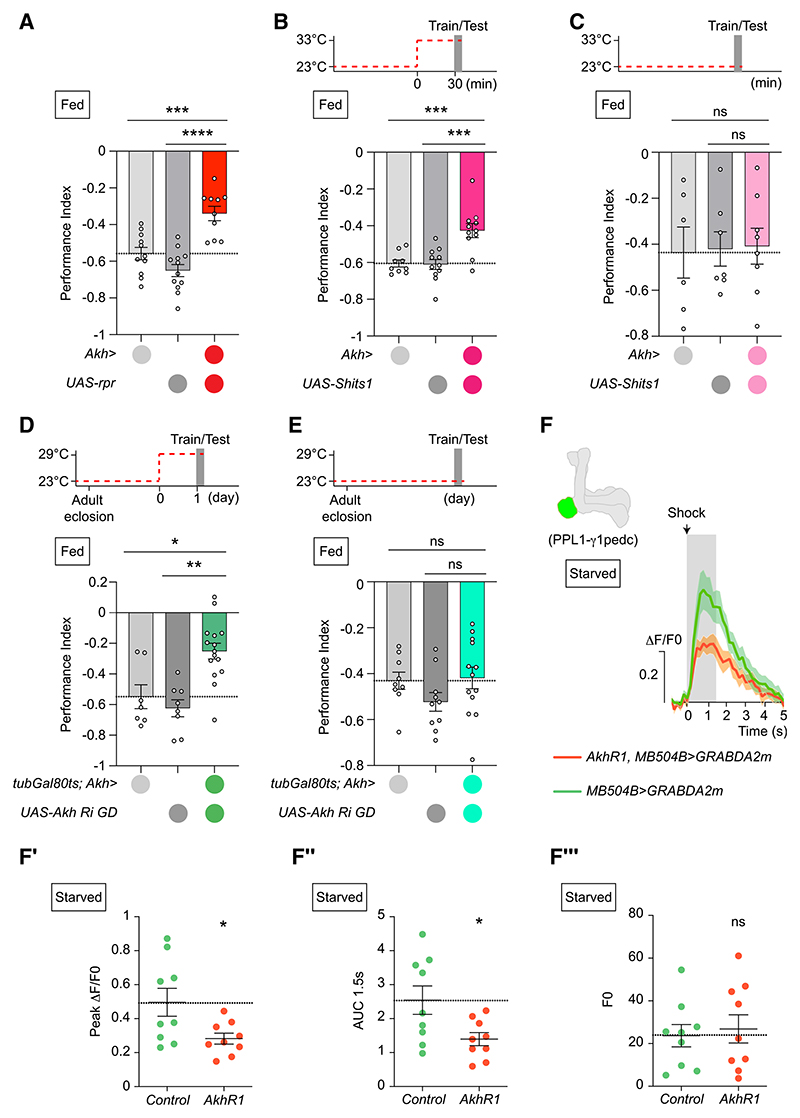
Corpora cardiaca cells and AKH are required for aversive learning (A) Corpora cardiaca (CC) ablation in *Akh>UAS-rpr* flies impairs immediate aversive memory, *n* ≥ 10. (B) Blocking CC secretion for 30 min (*Akh>*UAS-*Shi*^ts1^ flies) before and during training and testing impairs immediate aversive memory, *n* ≥ 9. Top: temperature shifting protocol. (C) Permissive temperature control for (B), *n* ≥ 6. Top: protocol. (D) *Akh* knockdown in adult CC impairs aversive immediate memory (*tubGal80^ts^*; *Akh>UAS-Akh Ri GD* flies), *n* ≥ 7. Top: temperature shifting protocol. (E) Permissive temperature control for (D), *n* ≥ 9. Top: temperature shifting protocol. (F) Mean shock-evoked (90 V) ΔF/F_0_ dopamine transients measured *in vivo* from PPL1-γ1pedc DANs of hungry wild-type and *AkhR1* mutant flies, *n* = 9. UAS-*GRAB*_*DA2m*_ driven by MB504B-split-GAL4. Gray rectangle marks 1.5 s shock presentation. (F′) Quantification of peak ΔF/F_0_ dopamine transients in (F). (F″) Quantification of mean area under the curve calculated during 1.5 s shock of recordings in (F). (F‴) Quantification of baseline GRAB_DA2m_ values, F_0_, in wild-type and *AkhR1* mutant flies in (F). Data presented as mean ± SEM. Individual data points of behavioral graphs represent independent groups of approximately 200 flies. Asterisks denote significant differences: *p < 0.05, **p < 0.01, ***p < 0.001, ****p < 0.0001. See also Figure S2.

*Drosophila* AKH is exclusively produced by CC cells from late embryo to adulthood.^5^ CC peptides are secreted from dense-core vesicles in axon-like projections onto vascular, gut, and brain targets.^5,8,30–32^ We tested a role for CC cells in aversive learning using expression of an upstream activating sequence (UAS)-transgene encoding the pro-apoptotic gene *reaper* (*rpr*). CC ablation impaired immediate aversive memory (Figure 2A). Blocking dynamin-dependent secretion from CC, using a temperature-sensitive *Shibire*^ts1^ transgene,^33^ also impaired aversive learning at restrictive, but not permissive, temperature (Figures 2B and 2C). CC cells produce Limostatin (Lst) in addition to AKH.^34^ We therefore selectively reduced AKH in the CC using temporally restricted expression of two *Akh* transgenic RNA interference (RNAi) lines. Both *Akh* RNAi transgenes significantly reduced *Akh* mRNA in whole adult flies (Figures S2A and S2B). We confined *Akh* knockdown to adulthood using a constitutively expressed temperature-sensitive GAL80,^35^ an inhibitor of GAL4 transcription factor. Consistent with *Akh^AP^* and *AkhR^1^* nulls, *Akh* knock-down in adult CC (at GAL80 restrictive 33°C) compromised immediate aversive memory (Figures 2D and S2C). Moreover, no defect was evident if the same flies were processed at permissive 23°C, where RNAi expression should be off (Figures 2E and S2D). Furthermore, adult-restricted *Lst* knockdown in CC did not affect aversive memory (Figures S2E and S2F).

We also used *AkhR^1^* and the GRAB_DA_ sensor to test a requirement for AKH signaling in hunger-driven elevation of shock-evoked DA release from PPL1-γ1pedc DANs. Shock-evoked GRAB_DA_ responses were decreased in hungry *AkhR^1^* mutant over those in hungry wild-type flies (Figures 2F–2F‴). Consistent with satiated and hungry fly recordings (Figures S1G–S1G″), odor-evoked DA release did not differ between hungry wild-type and *AkhR^1^* flies (Figures S2F–S2F‴). Physiological and behavioral data therefore indicate AKH/AKHR-dependent signaling specifically alters shock-evoked PPL1-γ1pedc DA release and levels of aversive learning.

### AKHR-expressing neurons in the SEZ regulate aversive learning

**Figure 3:**
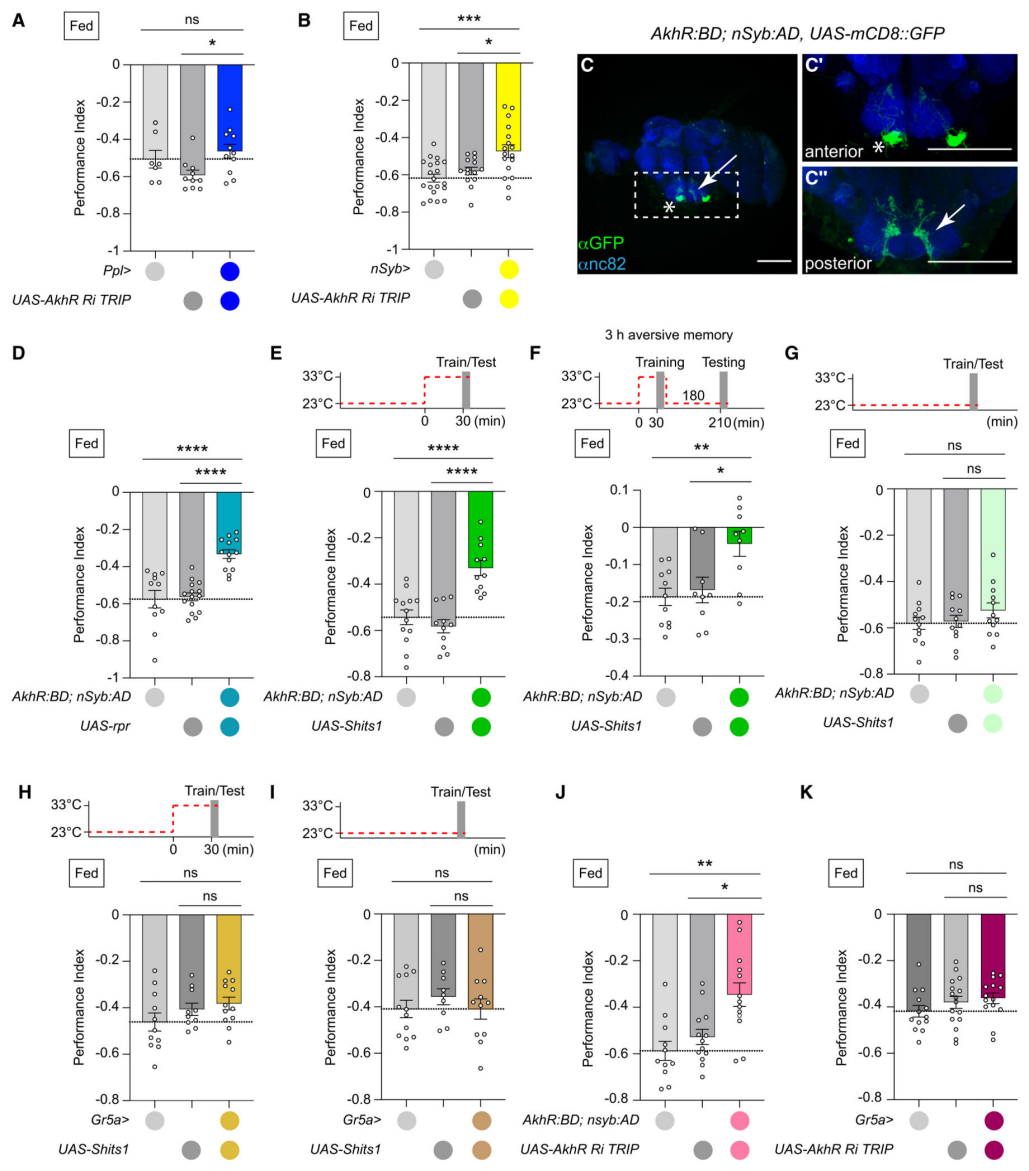
*AkhR* is required in subesophageal zone neurons for aversive learning (A) Silencing *AkhR* expression in fat body does not alter aversive learning (*ppl>AkhR Ri TRIP* flies), *n* ≥ 7. (B) Pan-neuronal *AkhR* knockdown impairs immediate aversive memory (*nSyb>AkhR Ri TRIP* flies), *n* ≥ 14. (C) *AkhR-*split-*GAL4* drives UAS-*mCD8::GFP* (green) in two neurons per hemisphere in the SEZ (AKHR neurons, asterisk) and in Gr5a-expressing GRNs (arrow). Neuropil generally stained with anti-nc82 (blue). (C′) Magnified anterior confocal projection showing AKHR neurons (asterisk). (C″) Magnified posterior confocal projection showing Gr5a GRN axons (arrow). (D) AKHR neuron ablation impairs immediate aversive memory (*AkhR::BD; nSyb::AD>UAS-rpr* flies), *n* ≥ 11. (E) Blocking AKHR neurotransmission during training decreases immediate aversive memory (*AkhR::BD; nSyb*::*AD*>*UAS*-*Shi*^ts1^ flies), *n* R11. Top: temperature shifting protocol. (F) Blocking AKHR output during training impairs 3 h aversive memory (*AkhR::BD; nSyb*::*AD*>*UAS*-*Shi*^ts1^ flies), *n* ≥ 9. Top: temperature shifting protocol. (G) Permissive temperature control for (E) and (F), *n* ≥ 12. Top: temperature shifting protocol. (H) Blocking Gr5a transmission during training did not alter aversive learning (*Gr5a>UAS-Shi*^ts1^ flies), *n* ≥ 11. Top: temperature shifting protocol. (I) Permissive temperature control for (H), *n* ≥ 9. Top: temperature shifting protocol. (J) *AkhR* knockdown in AKHR neurons impairs immediate aversive memory (*AkhR::BD; nSyb::AD>AkhR Ri TRIP* flies), *n* ≥ 11. (k) *AkhR* knockdown in Gr5a neurons did not alter immediate aversive memory (*Gr5a>AkhR Ri TRIP* flies), *n* ≥ 13. Data presented as mean ± SEM. Individual data points represent independent groups of approximately 200 flies. Asterisks denote significant differences: *p < 0.05, **p < 0.01, ***p < 0.001, ****p < 0.0001. Scale bars, 97 μm. See also Figures S3 and S4.

AKHR is a G-protein-coupled receptor. Reporter genes driven by *AkhR*-GAL4 show *AkhR* expression to be mainly, but not exclusively, in fat body and brain.^9,10,28,36–39^ To decipher AKHR’s site of action in aversive learning, we performed behavioral experiments on flies expressing *AkhR-RNAi* in the fat body using *pumpless* (*ppl*)-GAL4 and *collagen 25C* (*cg*)-GAL4, or pan-neuronally using *neuronal* Synaptobrevin (*nSyb*)-GAL4. Fat-body *AkhR* knockdown had no effect (Figures 3A, S3A, and S3B), whereas neuronal *AkhR* silencing impaired aversive memory (Figures 3B, S3C, and S3D). Two of the three behaviorally effective *AkhR* RNAi constructs produced significant decrease of whole fly *AkhR* mRNA, when driven in brain and fat body (with AKHR-GAL4); the other construct reduced levels but not significantly (Figures S3E–S3G). These results suggest metabolic and behavioral AKH/AKHR functions are supported by different organs.^10^

To characterize the neuronal locus of AKH action in aversive learning, we used intersection between halves of split-GAL4; *AkhR*-driven DNA binding domain (*AkhR:BD*) and pan-neuronal *nSyb*-driven activation domain (*nSyb:AD*).^10^ This combination drove GFP transgene expression in 2 neurons per hemisphere in the SEZ (asterisk) (Figures 3C and 3C′). The SEZ is the first gustatory processing center in the brain and also receives ascending signals via the ventral nerve cord (VNC). As previously described, *AkhR* expression was also evident in sweet-sensing gustatory receptor neurons (*Gr5a* GRNs, arrow) (Figures 3C and 3C″) and in VNC neurons commonly labeled with *Gr5a*-GAL4 (Figures S3H and S3I).^9^ From here, we refer to *AkhR:BD*; *nSyb:AD*>10xUAS-mCD8::GFP labeled neurons as AKHR neurons.

To verify that AKHR neurons express functional AKHR, we applied AKH peptide to exposed brains of live flies expressing GCaMP7f^40^ with *AkhR:BD; nSyb:AD*. Bath applied 1 nM AKH evoked a robust calcium response in AKHR neurons (Figure S3J), even in the presence of 100 nM tetrodotoxin (TTX) (Figure S3K), suggesting that AKHR neurons are directly AKH responsive. Importantly, AKHR neuron responses to AKH were not evident in *AkhR1* mutant brains, although they responded to 30 mM KCl application (Figure S3L). Lastly, CaLexA labeling revealed a robust hunger-driven increase in AKHR neuron activity (Figure S3M), consistent with the expectation that hunger-evoked CC release of AKH increases AKHR neuron activity through AKHR engagement.

We next assessed the functional relevance of AKHR neurons in aversive learning. Both ablation of (Figure 3D), and blockade of dynamin-dependent output from, AKHR neurons during training decreased aversive learning and 3 h memory (Figures 3E and 3F; no effect was observed at permissive 23°C, Figure 3G). Moreover, flies with disrupted AKHR neurons displayed normal shock and odor acuity (Figures S4A–S4D). Selectively blocking output from *Gr5a*-GAL4 neurons did not affect aversive learning (Figures 3H and 3I). Blocking AKHR neurons did not impair sugar-reward learning, consistent with performance of *Akh/AkhR* flies (Figures S4E and S4F).

To more firmly link AKH neuronal signaling to aversive learning, we drove *AkhR* RNAi in AKHR neurons using *AkhR* split-GAL4. Flies with *AkhR* knockdown in AKHR neurons exhibited reduced aversive learning (Figures 3J, S4G, and S4H), comparable to flies with pan-neuronal *AkhR* knockdown (Figures 3B, S3C, and S3D). *Gr5a*-GAL4-driven *AkhR* RNAi did not impair aversive learning (Figure 3K). Therefore, AKH signaling through AKHR neurons is required for aversive learning.

### AKHR neurons are presynaptic to neurons ascending to superior brain neuropils

**Figure 4:**
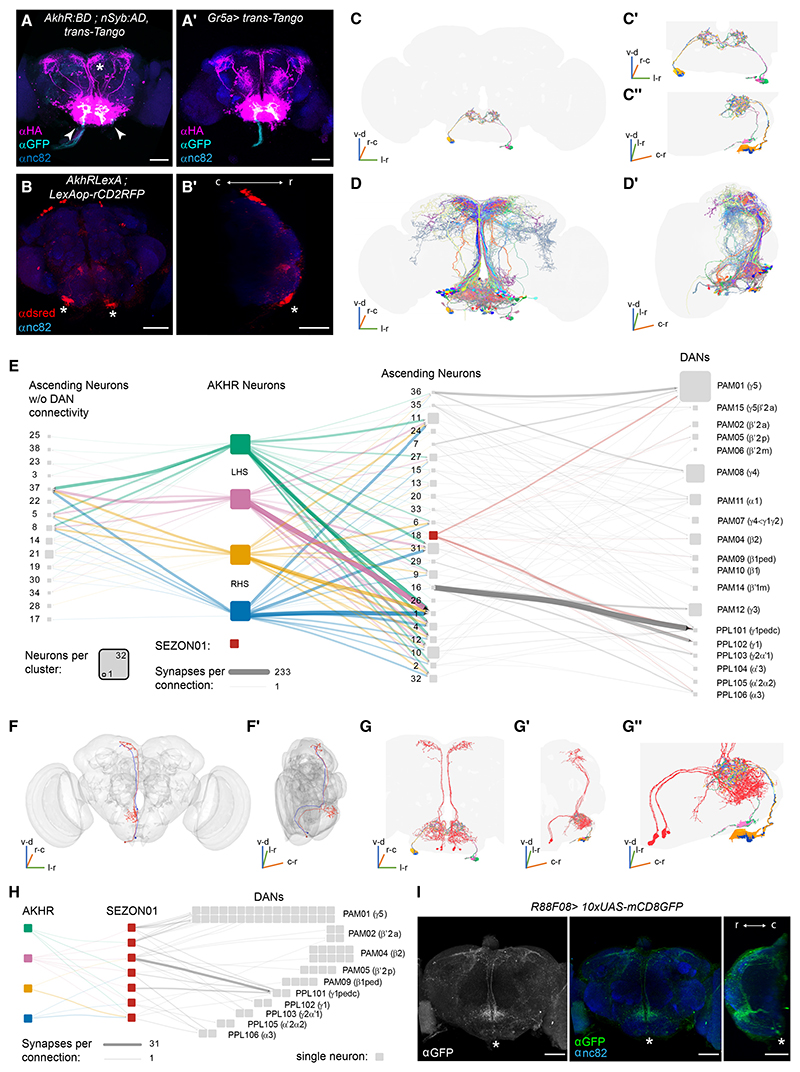
AKHR neurons connect to DANs via ascending SEZ output neurons (A) *trans*-Tango tracing of AKHR neurons labels neurons ascending to superior brain neuropils (*AkhR::BD; nSyb::AD>UAS-myrGFP.QUAS-mtdTomato-3xHA* flies). AKHR and Gr5a neurons labeled with anti-GFP (cyan) and *trans*-Tango signal with anti-hemagglutinin (HA) (magenta). Asterisk marks axons ascending into middle of SMP labeled downstream of AKHR (A) but not Gr5a neurons (A′). Brain neuropil stained with anti-nc82 (blue). (A′) *trans*-Tango labeling of Gr5a downstream neurons (*AkhR::BD; nSyb::AD>UAS-myrGFP.QUAS-mtdTomato-3xHA* flies) identifies many ascending neurons similar to those downstream of AKHR neurons in (A). Gr5a neurons labeled with anti-GFP (cyan) and *trans*-Tango signal with anti-HA (magenta). Brain visualized with anti-nc82 (blue). (B and B′) Frontal and lateral views of *AkhR-LexA*>rCD2::RFP (red) labeling of AKHR neurons confirm the unique morphologies of the 2 neurons in each hemisphere with large, oddly shaped cell bodies (asterisks) at the root of the pharyngeal nerve. Brain neuropil stained with anti-nc82 (blue). (C–C″) Frontal and lateral views of 3D representations of the four AKHR (aka ISN) neuron reconstructions from the FAFB dataset obtained from FlyWire via CAVEclient. Morphologies are unique and match those seen in (B). (D and D′) Frontal and lateral views of AKHR downstream neurons ascending to the superior brain through tracts like those seen with *trans*-Tango in (A). (E) Diagram showing connectivity between AKHR neurons, clusters of downstream ascending neurons, and DANs. Individual AKHR neurons shown in different colors to highlight connectivity patterns to ascending neurons. DANs clustered according to hemibrain type. Node size reflects number of neurons in each cluster. Edge size and opacity reflects number of synapses per connection, ranging 1–233. Ascending neuron clusters sorted for legibility of DAN connectivity, and DANs sorted by function/valence. SEZON01 ascending neurons (cluster 18, red) connect to PPL1 and PAM DANs. (F and F′) Frontal and lateral views of manual tracing of neuronal backbones with mid-SMP axons from AKHR neurons *trans*-Tango in (A) (blue) compared with morphology of SEZON01 skeletons produced from FAFB electron microscopy (EM) dataset (red^42^), identified as best fit across FAFB skeletons through NBLAST. (G–G″) Frontal and lateral views of 3D representations of AKHR neurons and all 7 SEZON01 downstream of AKHR neurons. (H) Connectivity diagram showing all SEZON01 receiving inputs from AKHR neurons and connecting to single PAM and PPL1 DANs. All, but one, SEZON01 connect to PPL101 DANs, but only a subgroup also connects to PAM01 DANs. Edge size and opacity reflects synapse number per connection ranging 1–31. (I) R88F08-GAL4-driven UAS-mCD8::GFP (shown in white on the left and green in the middle) labels SEZON01 and potentially neurons from AKHR neurons downstream clusters 14 and 21 that are not connected to DANs. Brain neuropil visualized with anti-nc82 (blue). Right: lateral view showing distinct soma tracts of R88F08 neurons (green). Cell bodies marked (asterisk). Scale bars, 50 μm. See also Figure S5.

To understand how AKHR neurons participate in aversive learning, we determined their neuronal connectivity. We first used *trans*-Tango anterograde transsynaptic tracing to reveal neurons postsynaptic to AKHR neurons.^41^ This approach labeled neurons mainly in the SEZ, some of which ascend to superior brain regions via midline and other tracts (Figure 4A). Since *AkhR*-GAL4 also expresses in Gr5a GRNs, we independently performed *trans*-Tango with *Gr5a*-GAL4 (Figure 4A′). Neurons downstream of Gr5a neurons generally resembled those labeled with AKHR *trans*-Tango, with ascending neurons visible in both downstream populations. However, closer inspection revealed AKHR *trans*-Tango to label additional ascending neurons in midline tracts that have distinctive axon branching into the superior medial protocerebrum (SMP) (Figure 4A, asterisk).

To study downstream connectivity in detail, we identified AKHR neurons in the published transmission electron microscope volume of a full adult female brain (FAFB).^43^ AKHR neurons were easily recognizable in the FAFB dataset using the FlyWire platform based on their uniquely localized somata and neurite morphology (flywire.ai^44^), and we also computationally^45^ verified that they match those labeled with *AkhR* split-GAL4 (Figures S5A–S5C). Lastly, we excluded the existence of other FAFB neurons with similar morphology by comparing a trace of *AkhR*-Gal4 labeling AKHR neurons with FAFB AKHR neurons and their most similar neurons across the FAFB dataset using NBLAST^46^ (Figure S5C; Video S1; Table S1).

We noted that AKHR neuron somata lie alongside the pharyngeal nerve and are separated by a glial sheath from the brain (Figures 4B and 4C). Light microscopy confirmed that co-labeled AKHR neurons are external to the glial sheath, marked with antirepo staining, which labels glial nuclei (Figure S5D), or with R85G01-GAL4; UAS-GFP, which expresses in perineural glia (Figure S5E). AKHR neurons have also been called interoceptive SEZ neurons (ISNs)^38^ and are accordingly annotated as ISNs in FlyWire.^44^

We next identified neurons downstream of AKHR neurons and filtered those for individuals predominantly receiving inputs in the SEZ and projecting to superior brain neuropils close to the MBs (Figure 4D). This process retrieved 135 neurons that were subsequently clustered into 38 morphological groups containing 1–11 neurons each. In general, neuronal morphologies resembled those observed with *trans*-Tango (Figures 4A and 4D; Video S2). Most neuron clusters are postsynaptic to all 4 AKHR neurons and receive equal numbers of synapses from each neuron, perhaps expected given the tightly interwoven and bi-hemispheric nature of AKHR neuron axons.

### Ascending neurons connect AKHR neurons to reinforcing DANs

Analyzing downstream targets revealed 24 of 38 ascending neuron clusters are presynaptic to a collection of MB-innervating DANs with dendrites in superior neuropils (Figure 4E). Strength (inferred from the number of synapses) of the connections ranged from a single synapse from one of three neurons in cluster 1 to an individual PAM11 (α1) DAN, to 233 synapses between the two neurons (one per hemisphere) in cluster 16 to both PPL101 (γ1pedc) DANs. Cluster 16 neurons also connect to other DAN types. Interestingly, whereas three neurons in cluster 1 are the strongest downstream ascending neuron target of all 4 AKHR neurons, cluster 16 receives relatively little AKHR neuron input. This reciprocal pattern of connectivity from AKHR neurons and to DANs applies to all ascending clusters. Those strongly connected to AKHR neurons are weakly connected to DANs, and vice versa.

All ascending neuron clusters most strongly connect to the PPL101 (γ1pedc), PPL102 (γ1), and PPL106 (α3) DANs as well as PAM01 (γ5), PAM04 (β2), PAM08 (γ4), PAM11 (α1), and PAM12 (γ3), and with fewer synapses to PAM15 (β′2γ5) and the β′2 DANs (PAM02-β′2a, PAM05-β′2p, and PAM06-β′2m). Importantly, some ascending neuron clusters co-wired to DANs of similar functionality/valence, e.g., aversively reinforcing PPL101 (γ1pedc), PPL102 (γ1), PPL106 (α3), and PAM12 (γ3) DANs (Figure 4E), as previously observed.^42,47^ Connectomics therefore indicate that AKHR neurons are positioned to modulate the activity of ascending pathways that provide input to reinforcing DANs.

### SEZON01 ascending neurons link AKHR neurons to aversive PPL101(γ1pedc) DANs

Both *trans*-Tango and connectomics with AKHR neurons identified postsynaptic ascending neurons with a dorsal soma tract and that project axons through the midline into the SMP (Figures 4A, 4A′, and 4D; Video S1). We therefore manually traced a rudimentary backbone of these midline neurons within the *trans*-Tango AKHR pattern. Then, using NBLAST,^46^ we compared the traced backbone with skeletons of a population of SEZ output neurons (SEZONs) of similar morphology, which we previously reconstructed and identified in FAFB^42^ (Figure 4F). These neurons are now available in FlyWire, and further alignment retrieved SEZON01 neurons as being in cluster 18 of the AKHR downstream neurons^42,47^ (Figure 4G). SEZON01 axons project in a midline bundle then arborize in the SMP where they synapse onto PPL101 (γ1pedc), PAM01 (γ5), and PAM02 (β′2a) DANs (Figure 4H).

We found that 6 of 7 SEZON01 neurons that were downstream of AKHR neurons connected to PPL101 (γ1pedc) DANs. Three of these SEZON01s also synapsed onto a particular subtype of PAM01 (γ5) DANs. Since PPL101 DANs are required to form short-term aversive olfactory memories reinforced with electric shock, bitter taste, or noxious heat,^23,24,48,49^ we considered SEZON01 neurons to be good candidates to link AKHR neurons to DANs involved with aversive learning.

### AKHR-SEZON01-PPL101 neuronal circuit is generally required for aversive learning

**Figure 5:**
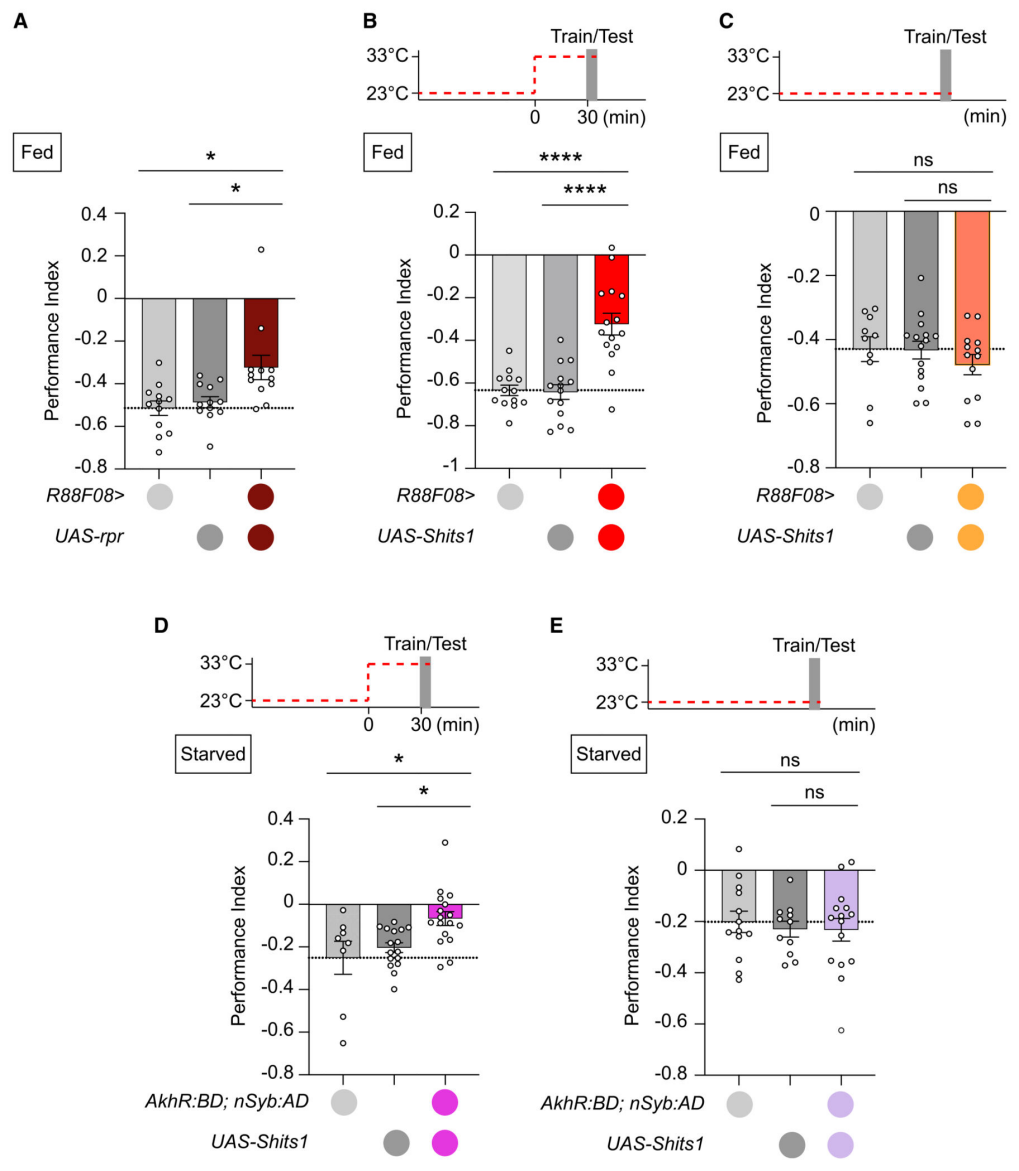
SEZON01 and AKHR neurons are generally required for aversive learning (A) SEZON01 ablation impairs immediate shock-reinforced aversive memory (*R88F08>UAS-rpr* flies), *n* ≥ 12. (B) Blocking SEZON01 neurotransmission for 30 min before and during odor-shock learning reduces immediate aversive memory (*R88F08>UAS-Shi*^ts1^ flies), *n* ≥ 13. Top: temperature shifting protocol. (C) Permissive temperature control for (B), *n* ≥ 10. Top: temperature shifting protocol. (D) Blocking AKHR neurotransmission for 30 min before and during odor-bitter learning abolishes immediate aversive memory (*AkhR::BD; nSyb::AD>UAS-Shi*^ts1^flies), *n* ≥ 8. Top: temperature shifting protocol. (E) Permissive temperature control for (D), *n* ≥ 11. Top: protocol. Data presented as mean ± SEM. Individual data points represent independent groups of approximately 200 flies. Asterisks denote significant differences: *p < 0.05, ****p < 0.0001. See also Figure S6.

We previously described GMR88F08-GAL4 (from here, R88F08-GAL4) to label SEZON01 neurons.^42^ In addition, pairing odor exposure with optogenetic SEZON01 activation produced aversive odor memory, while their blockade impaired bitter-taste reinforced aversive learning. We here noted that R88F08-GAL4 (Figure 4I) expresses in SEZON01 neurons and in potentially two other ascending neuron clusters, 14 and 21, downstream of AKHR neurons. However, neither 14 nor 21 clusters connect to DANs. We therefore used R88F08-GAL4 to test whether SEZON01 were also required for shock-reinforced aversive memory. Flies in which SEZON01 were ablated or transmission was conditionally blocked exhibited reduced aversive learning (Figures 5A and 5B). Importantly, no defect was apparent at permissive 23°C (Figure 5C). In addition, odor and shock acuity of R88F08>UAS-*Shi*^ts1^ flies were comparable to controls at restrictive 33°C (Figures S6A–S6C). Therefore, SEZON01 are required to convey the reinforcing effects of electric shock in addition to bitter taste, suggesting they may generally relay aversive information to PPL101(γ1pedc) DANs. Since compromising SEZON01 neurons does not completely abolish aversive learning performance, we assume other ascending pathways contribute to relay shock and bitter input to aversively reinforcing DANs.

AKH/AKHR signaling also modulates bitter sensing upon starvation.^50^ We therefore tested whether AKHR neurons were also required for aversive learning reinforced with bitter taste, in addition to shock learning (Figures 3D and 3E). Blocking AKHR neurons with UAS-*Shi*^ts1^ abolished bitter (N,N-diethyl-meta-tol-uamide [DEET]) learning at restrictive (Figure 5D) but not permissive temperature (Figure 5E). Therefore, AKHR neurons, like SEZON01 neurons and PPL101 DANs, are required for both electric-shock- and bitter-taste-reinforced aversive learning.

### OA from two of the four AKHR neurons acts on SEZON01 neurons

Prior work concluded AKHR neurons to be octopaminergic based on detection of transcripts for *Tbh*, the gene encoding tyramine β-hydroxylase that turns tyramine into OA, in mRNA from pooled AKHR neurons.^10^ However, automated prediction of synaptic transmitter identity^44,51^ annotated AKHR neurons in FAFB as likely to be cholinergic, with some serotonergic-like synapses (Figures S7A–S7A‴). We also noted that AKHR neuron ultrastructure appears different to that of typical cholinergic and octopaminergic neurons (data not shown).

**Figure 6:**
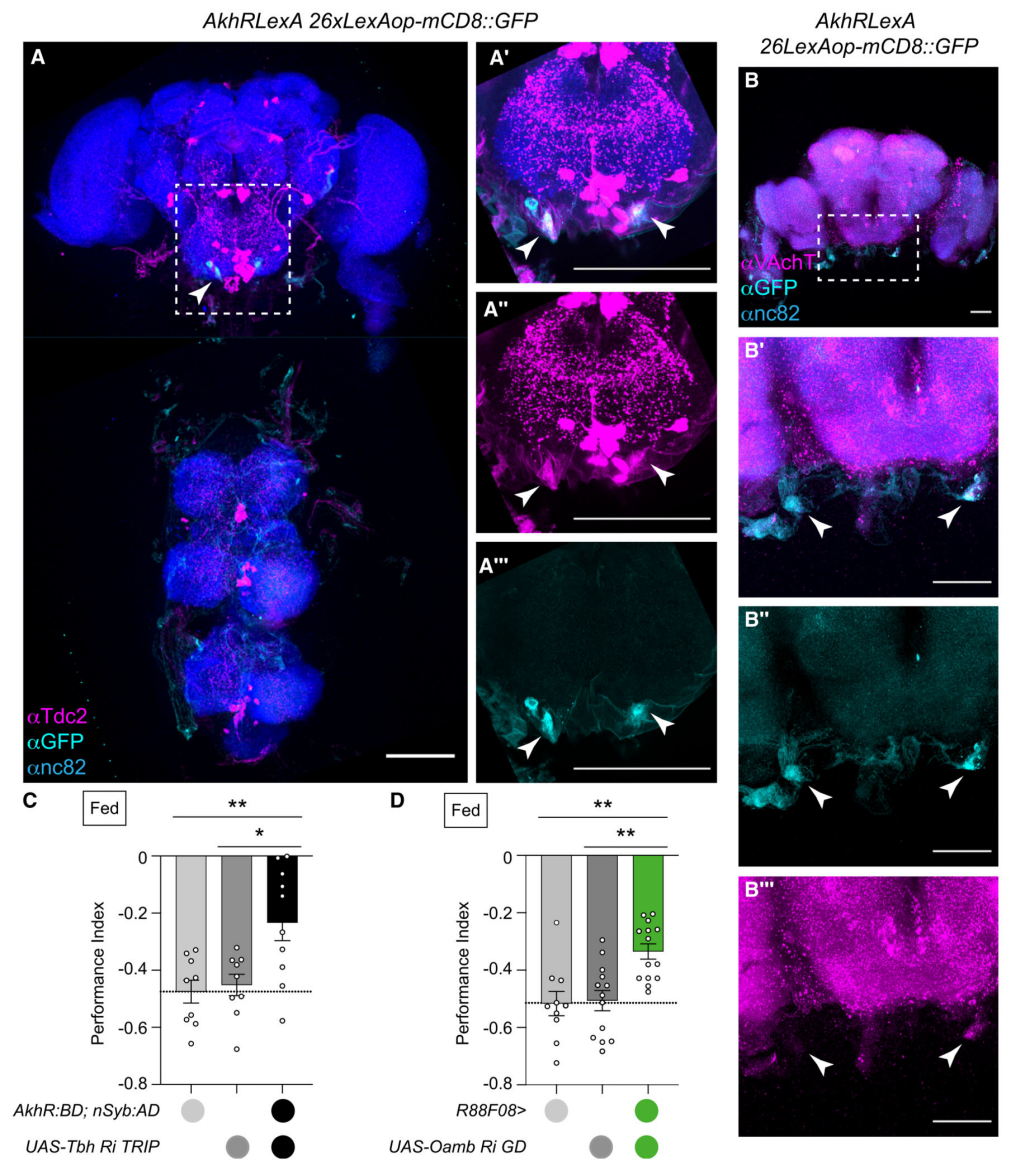
AKHR neurons are octopaminergic and require *T*β*h* for aversive learning (A) *AkhR*-LexA drives *26xLexAop-mCD8::GFP* (cyan) in four AKHR neurons. One *AkhR* neuron per hemisphere co-stained (white, arrowhead) with anti-Tdc2 (magenta). Brain neuropil visualized with anti-nc82 (blue). Scale bars, 88 μm. (A′–A‴) Magnified views of SEZ showing (A′) the merge, (A″) anti-Tdc2 staining (magenta), and (A‴) the AKHR neurons (cyan). Scale bars, 88 μm. (B) *AkhR-LexA>26xLexAop-mCD8::GFP* (cyan) neurons also co-stained with anti-VAchT (magenta) (arrowheads mark somata). Scale bars, 50 μm. (B′–B‴) Magnified views of SEZ showing (B′) merge, (B″) AKHR neurons (cyan), and (B‴) anti-VAchT staining (magenta). Scale bars, 50 μm. (C) *Tbh* knockdown in AKHR neurons impairs immediate shock-reinforced aversive memory (*AkhR::BD; nSyb::AD>Tbh Ri TRIP flies*), *n* ≥ 9. (D) *Oamb* knockdown in SEZON01 neurons impairs immediate shock-reinforced aversive memory (*R88F08>Oamb Ri GD* flies), *n* ≥ 10. Data presented as mean ± SEM. Individual data points represent independent groups of approximately 200 flies. Asterisks denote significant differences: *p < 0.05, **p < 0.01. See also Figure S7.

We therefore verified AKHR neurons as being octopaminergic by immunostaining for *Tdc2*-encoded tyrosine decarboxylase, which produces OA’s precursor tyramine from tyrosine. Surprisingly, only one of two AKHR neurons per hemisphere labeled with GFP reliably co-stained with anti-Tdc2^52^ (Figures 6A–6A‴), indicating the other two may not be octopaminergic. AKHR neurons also showed punctate labeling with anti-VAChT anti-body^53^ directed against vesicular acetylcholine transporter (Figures 6B–6B‴). However, signal to noise of the staining was too variable to determine whether all AKHR neurons are cholinergic.

We next tested whether OA production is required for AKHR neuron function in aversive memory. RNAi knockdown of *Tbh* in AKHR neurons impaired aversive memory compared with both genetic controls (Figure 6C), confirming AKHR neuron-produced OA is required for robust aversive learning.

To determine if aversive learning required OA downstream signaling in SEZONs, we knocked down expression of the *Oamb*,^54^ *Octα2R*,^55,56^ *Octα1R*,^57–59^ *Octα2R*,^57^ *Octα3R*,^57^ and *Oct-TyrR*^58^ encoded OA receptors with R88F08-GAL4-driven RNAi. Only knockdown of α-adrenergic-like OAMB receptor significantly impaired aversive learning (Figures 6D and S7B–S7F). Since OAMB activation is known to increase intracellular calcium^54,59^ its engagement would be predicted to bring SEZON01 neurons closer to threshold and therefore easier to trigger.

Considering AKHR neuron anti-Tdc2 immunostaining and *Tbh* RNAi data together with that from *Oamb* RNAi in SEZONs, we conclude that OA release from AKHR neurons acts through OAMB to modulate SEZON01 activity so that they can efficiently relay the reinforcing effects of shock to PPL1-γ1pedc DANs.

### The CC-AKHR-SEZON01-PPL101 axis mediates hunger-enhanced aversive learning

**Figure 7:**
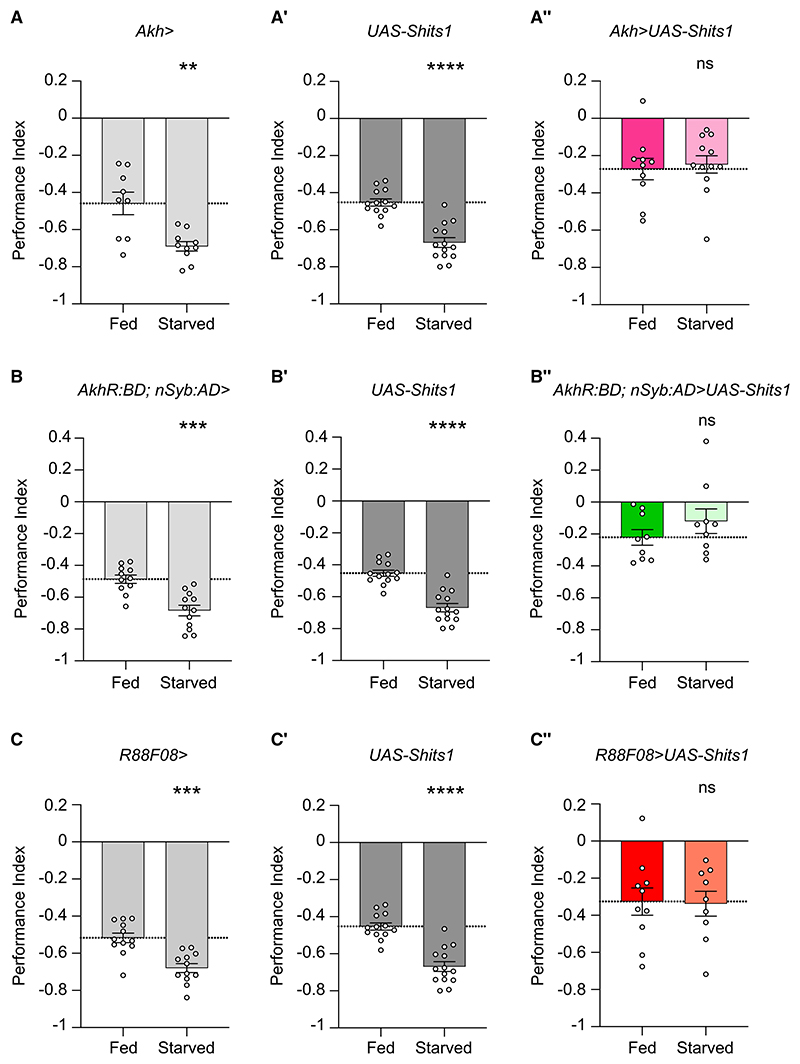
The CC-AKHR-SEZON01-PPL101 axis mediates hunger-enhanced aversive learning (A–A″) Immediate shock-reinforced aversive memory is enhanced by starvation in parental control flies (A and A′) but not when secretion is conditionally blocked from CC with UAS-*Shi*^ts1^ (A″), *n* ≥ 9. (B–B″) Immediate shock-reinforced aversive memory is enhanced by starvation in parental controls (B and B′) but not when transmission is conditionally blocked from AKHR neurons (B″), *n* ≥ 9. (C–C″) Immediate shock-reinforced aversive memory is enhanced by starvation in parental controls (C and C′) but not when transmission is conditionally blocked from SEZON01 (C″), *n* ≥ 9. Flies were shifted from 23°Cto 33°C30 min before and during training and testing. Data presented as mean ± SEM. Individual data points displayed as dots represent independent groups of approximately 200 flies. Asterisks denote significant differences: *p < 0.05, **p < 0.01, ****p < 0.0001. The *UAS-Shi*^*ts1*^ parental control data displayed in (A′), (B′), and (C′) are the same as they were collected in parallel with those from all other genotypes.

**Figure 8:**
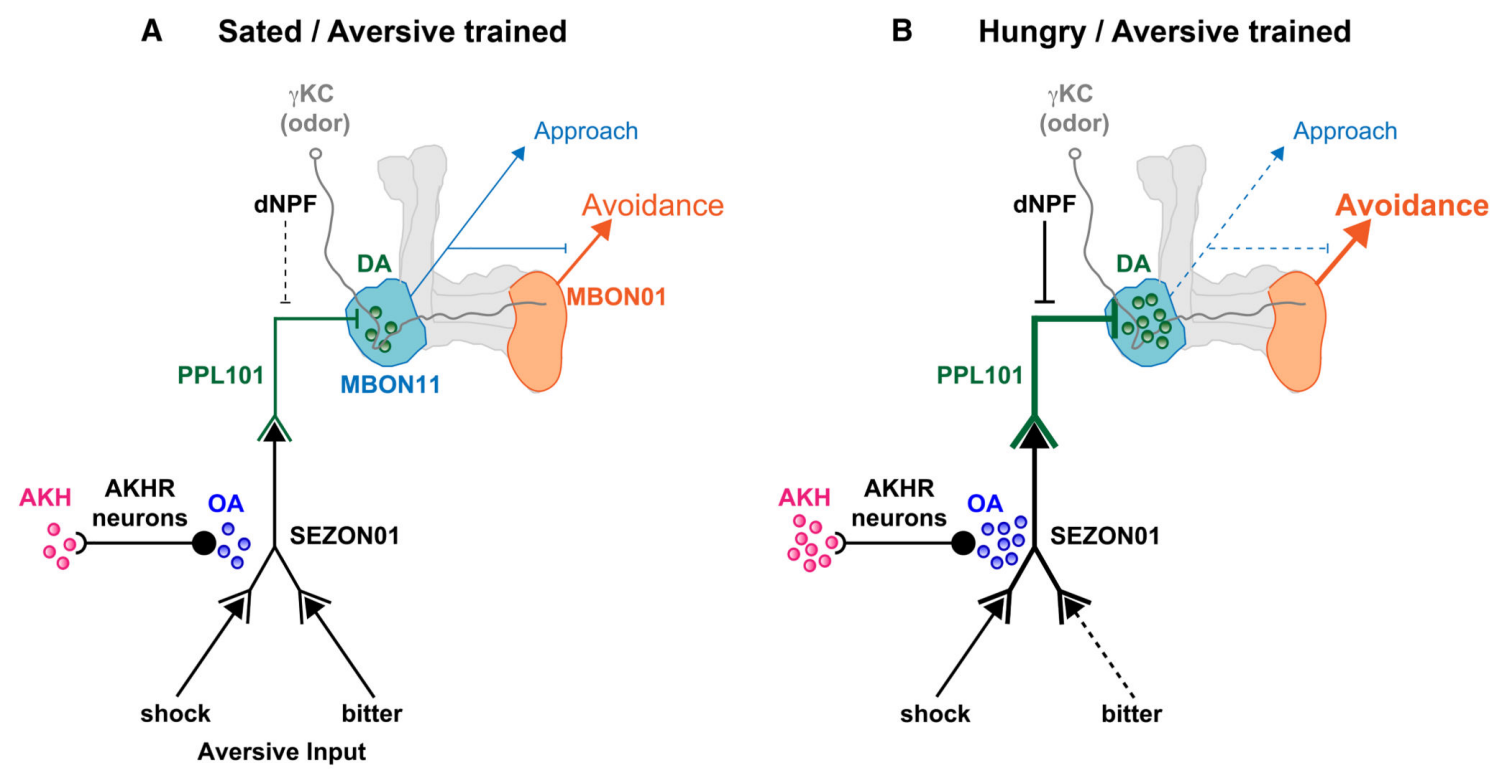
Model: Input enhancement maintains aversive reinforcement function (A) PPL101 (γ1pedc) DANs (green) have a dual function in appetitive motivation and reinforcement of aversive learning, with shock and bitter taste.^13,24,48^ Tonic dopamine release from PPL101 DANs suppresses the odor-driven excitability of the GABAergic approach-directing mushroom body output neuron (MBON11, blue) in food-satiated flies.^60^ In hungry flies elevated dNPF relieves the PPL101-mediated suppression, which elevates odor-driven MBON11-directed approach/ appetitive behavior.^13,60^ Phasic PPL101 activity is evoked by aversive stimuli^27,61^ and released dopamine writes aversive memories by depressing connections between odor-specific KCs and MBON11.^60,62^ This leaves other MBON outputs, such as avoidance-favoring MBON01 (orange) to direct conditioned odor avoidance behavior.^63,64^ PPL101 input SEZON01 neurons are required for aversive learning reinforced by shock and bitter taste, and SEZON01 are subject to OA-directed modulation from hunger-responsive AKHR neurons that respond to circulatory AKH even in the satiated condition. (B) When hungry, higher circulatory AKH stimulates AKHR neurons to release more OA onto SEZON01 neurons and other ascending pathways (data not shown). OA via OAMB receptor-coupled intracellular Ca^2+^ release lowers activation threshold of SEZON01 neurons so that shock and bitter-taste inputs (which are desensitized in hungry flies^50^) are more efficiently transduced to PPL101 DANs. Parallel AKH-dependent enhancement of SEZON01-PPL101 input thereby allows reinforcers to overcome the concurrent motivation-related dNPF-mediated suppression of tonic PPL101 firing, which maintains the fly’s ability to learn about aversive events when hungry. Line thickness, dashing, and arrowhead size denote relative activity levels of relevant neurons.

Having established roles for CC-AKHR-SEZON01 functional circuit elements in satiated-baseline aversive learning, we last tested whether they were critical for enhancement of aversive learning following chronic starvation. We again used UAS-*Shi*^ts1^ to block secretion from CC, the AKHR neurons, and SEZON01 and tested aversive learning in both fed and starved states in parallel. As expected, all starved parental control genotypes displayed enhanced aversive learning compared with fed flies of the same genotype (Figures 7A, 7A′, 7B, 7B′, 7C, and 7C′). By contrast, blocking output from CC, AKHR neurons, or SEZON01 abolished the starvation-induced enhancement of aversive learning—all groups displayed similar memory in both fed and starved conditions (Figures 7A″, 7B″, and 7C″). Therefore, CC, AKHR neurons, and SEZON01 block in starved flies recapitulated the phenotype obtained with starved *Akh* and *AkhR* mutants. These observations lead us to propose a model (Figure 8) in which circulatory AKH tunes the level of OA release from AKHR neurons. OA via the OAMB receptor enhances SEZON01 neuron excitability so they can be more efficiently driven by shock (and bitter taste), and their transmission in turn establishes the strength of the DA reinforcement signal from PPL1-γ1pedc DANs.

## Discussion

### AKH signaling adjusts input to aversively reinforcing DANs

Internal sensing of nutrient deficit triggers a systemic response that mobilizes energy stores and prepares animals for resource-seeking behaviors. Neuroendocrine release of circulatory hormones is a key element orchestrating these bodily responses.

As the insect analog of glucagon, release of AKH into the hemolymph plays a metabolic role in mobilizing carbohydrate and lipid stores during periods of energy deficit.^5,8,65^ AKH has also been assigned a role in inducing starvation-induced hyperactivity, which presumably facilitates food seeking.^5,10,29^ Expression of AKHR in 4 neurons in the fly’s SEZ suggests a very localized site of AKH action in the brain. However, beyond their production and release of OA, it was not known how AKHR neurons regulate behavior.

We found AKHR neurons connect to a dense network of SEZ neurons including an array of ascending neurons projecting axons into the dorsal brain that provide input to reinforcing DANs. AKH-dependent modulation of at least one of these ascending pathways, SEZON01, facilitates dopaminergic reinforcement of aversive learning when the fly is hungry. We propose one purpose of enhancing these inputs to aversively reinforcing DANs is to compensate for hunger-dependent suppression of their baseline activity by dNPF, which is necessary to promote expression of sugar-seeking memories.^13,60^ Recording DA release from PPL101 DANs showed both a hunger-dependent reduction of baseline DA (the effect of elevated dNPF) and enhancement of shock-evoked release (the consequence of elevated AKH). Coordinating suppression of PPL101 baseline activity with compensatory enhancement of input constitutes a form of network homeostasis that allows the hunger-dependent motivational role of the DANs to occur while maintaining reinforcer function when required.

Another plausible reason why hunger might facilitate input to aversively reinforcing DANs is to compensate for state-dependent reduction in strength of their own inputs. We found that AKHR neuron-directed modulation of SEZON01 neurons is required for aversive learning with both shock and bitter taste, as are PPL101 DANs,^48^ and that SEZON01 receive direct GRN input. Like other hungry animals, food-deprived flies tolerate more bitter taste in their food and risk ingesting potentially toxic substances to maximize calorie intake.^50,66^ Tolerance of bitter taste partly arises from AKH-dependent reduction in responsiveness of bitter-sensing Gr66a-expressing GRNs.^50^ Therefore, coordinated hunger-dependent facilitation of downstream SEZON01 could serve as network gain control for reduction in bitter GRN input. This would allow flies to preserve the ability to remember experiencing bitter-tasting food when risking its ingestion.

Although early studies established AKH to be secreted during energy deficit,^5,8,10,29^ we found AKH is also required for efficient aversive learning in sated flies. It therefore seems AKH signaling is more finely tuned than previously expected^67^ and that it constantly influences activity of ascending pathways to adjust behavioral and metabolic aspects of energy homeostasis. AKH release may also be under nutrition-independent control.^68–70^

Refeeding reversed hunger-dependent enhancement of aversive learning, demonstrating the facilitating process is dynamic. Other neuropeptides might be involved in the reversal, although *Drosophila* insulin-like peptide 3 (Dilp3) was implicated in maintenance but not acquisition of aversive memory.^71^

### AKHR neurons modulate downstream circuits using OA

Our results and those of others^36,38,65,72^ demonstrate that AKH activates AKHR neurons. We found AKHR neuron somata are outside the glial sheath surrounding the brain. This positioning resembles osmosensory neurons in mammals, which have processes outside the blood-brain barrier^73^ and suggests that AKHR neurons have access to the nutrient composition of the fly’s circulatory hemolymph and to endocrine hormones such as AKH.

Our work questions whether all four AKHR neurons are octopaminergic.^10^ In addition, since most octopaminergic neurons in the brain typically co-express markers for glutamate co-release,^74^ AKHR neurons appear to be atypical octopaminergic neurons. Although morphology and upstream and downstream connectivity of the two AKHR neuron pairs are similar, they may use different neurotransmitters. At this stage, we can only conclude that OA synthesis in, and presumably release from, at least two of the AKHR neurons is critical for aversive learning. We postulate that hunger-scaled levels of circulatory AKH have a graded effect on the activity of AKHR neurons, which in turn results in a graded “tone” of OA released onto dendrites of ascending neurons.

### Aversive learning requires OAMB function in ascending SEZON01 neurons

We also demonstrated that the α-adrenergic-like OAMB receptor is required in SEZON01 for aversive learning with shock, consistent with AKHR neuron-released OA modulating SEZONs via this receptor. Since OAMB couples to calcium release from intracellular stores,^54,59^ we propose that hunger-scaled OAMB-directed modulation of SEZON01 dendrites brings them closer to threshold (Figure 8) and therefore more sensitive to being triggered by their upstream input neurons, such as those relaying shock from the legs. Since AKHR neurons, SEZON01, and PPL1-γ1pedc DANs are also required for bitter learning, we predict this is likely to also apply to SEZON01 inputs from bitter-taste GRNs in the proboscis. Our analysis of connectivity in FlyWire revealed 65 neurons annotated as GRNs to be upstream of 6 SEZON01 neurons. Individual GRN-SEZON01 connections ranged from 1 to 21 synapses. Importantly, we did not find any GRNs to be directly upstream of AKHR neurons. Connectivity therefore suggests AKHR neurons are not direct conduits of shock and bitter aversive signals but instead facilitate, e.g., GRN-SEZON01 synaptic efficacy in a heterosynaptic manner via post-synaptic modulation of SEZON01 dendrites (Figure 8). Testing this model further will require recordings of sub and suprathreshold events from SEZON01 neurons. Unfortunately, R88F08-GAL4-driven expression was too weak to visualize or live-image these neurons *in vivo*.

Although multiple roles in *Drosophila* appetitive learning have been elucidated for OA signaling,^48,54,75–77^ studies of aversive learning in *TβH*^*18M*^ mutants lacking OA were contradictory.^75,78^ Our data support Iliadi et al.^78^ and show a role in shock-rein-forced aversive learning for OA production in AKHR neurons and OAMB in SEZON01 neurons.

### What are other AKHR post-synaptic ascending pathways doing?

Although our functional analyses focused on SEZON01 neurons, connectomics revealed 23 of 38 other ascending clusters to also connect to DANs and 15 clusters that do not. We assume AKHR neurons modulate activity of all of these ascending pathways and that they could, for example, as a whole signal a DAN-dependent aversive state of hunger, or “cost on inaction.”^79^ A recent study demonstrated a role for the AKHR/ISN neuron-connected bilateral T-shaped (BiT) neurons that connect to neuroendocrine centers in differentially regulating water and sugar ingestion.^80^ It will be interesting to determine roles of other ascending pathways and whether they are facilitated or inhibited by AKHR neuron-released OA. Detailed analysis of input and output connectivity of ascending neurons might be informative of possible functions. However, further neuronal typing and new genetic reagents (such as those in Sterne et al.^81^) are needed to independently manipulate and record from different ascending neurons.

### Could there be a similar modulation of DAN input in vertebrates?

The most obvious functional parallel in mammals for the AKHR-DAN ascending neurons we describe in the fly is in connections between nutrient deficit-responsive neurons in the hypothalamus, such as Agouti-related peptide (AGRP) neurons, and DANs in the ventral tegmental area (VTA). Studies have, for example, shown that activation of AGRP neurons,^79^ or of a glutamatergic lateral hypothalamus (LH)-VTA projection^82^ mediates aversion, whereas activation of GABA-ergic LH-VTA neurons promotes reward.^82^

Surprisingly, α1-adrenergic receptors, which—like the fly OAMB receptor—gate release of intracellular Ca^2+^, have been reported to inhibit GABA-ergic inputs to VTA but stimulate the glutamatergic inputs.^83^ Such coordinated action should thereby potentiate aversive reinforcement, like OA through OAMB in SEZON01 and other ascending neurons in the fly. LH-VTA pathways are also responsive to nutritional state and food reward via numerous classes of peptidergic neurons in LH. Moreover, more than half of LH-VTA neurons express GLP-1R, the receptor for glucagon-like peptide 1,^84^ which also responds to glucagon at lower affinity.^85^

Interestingly, GLP-1 and GLP-1R have been independently implicated in aversive memory enhancement in both a passive avoidance and water maze task.^86^ By contrast, GLP-1R activation in LH attenuates food and drug reinforcement.^87–89^ Lastly, hypothalamic expression of exendin-4, a natural and uncleavable GLP-1R agonist from the toxin of the Gila monster lizard (and synthetic variants of it), has been reported to restore DAN functions in Parkinson’s disease models in rodents, even after damage is established.^90,91^ One plausible explanation for restorative function could be that, like AKH in the fly, GLP-1/exendin-4 alters the activity of input pathways to the DAN populations that remain functional in the diseased brain.

## Star★Methods

### Key Resources Table

**Table:** 

REAGENT or RESOURCE	SOURCE	IDENTIFIER
Antibodies		
Chicken polyclonal anti GFP	abcam	Cat#ab13970; RRID: AB_300798
Rat monoclonal anti HA	Roche, Sigma Aldrich	Cat#3F10; RRID: AB_10094468
Mouse monoclonal anti nc82	DSHB	Cat#nc82; RRID: AB_2314866
Rabbit polyclonal anti dsred	Clontech	Cat#632496; RRID:AB_10013483
Rabbit polyclonal anti Tdc2	abcam	Cat#ab128225; RRID: AB_11142389
Goat polyclonal anti VachT	Sigma Aldrich	Cat#ABN100; RRID:AB_2630394
Mouse monoclonal anti Repo	DSHB	Cat#8D12; RRID:AB_528448
Chemicals, peptides, and recombinant proteins		
4-methylcyclohexanol (MCH)	Sigma Aldrich	Cat#66360
3-octanol (OCT)	Sigma Aldrich	Cat#218405
Mineral oil	Sigma Aldrich	Cat#M5904
Drierites	ThermoFisher	Cat#219040020
DEET	Fisher Scientific, MP Biomedicals	Cat#203013
Triolein	Sigma Aldrich	Cat#T7140
Paraformaldehyde 20%	Electron Microscopy Sciences	Cat#15713-S
Normal goat serum	Sigma Aldrich	Cat#G6767
Normal donkey serum	Sigma Aldrich	Cat#D9663
Vectashield	Vector Labs	Cat#H-1000
KCl	Sigma Aldrich	Cat#60142
Tetrodotoxin citrate	Abcam	Cat#ab120055
AKH synthetic peptide Glp-LTFSPDW-NH2	Sigma Aldrich	This study
Critical commercial assays		
Macherey-Nagel™ Nucleospin™ Tissue	Fisher Scientific	Cat#740952.50
Platinum™ SupeFi II PCR Master Mix	ThermoFisher Scientific	Cat#12368010
pENTR/D-TOPO cloning kit	ThermoFisher	Cat#K240020
Gateway™ LR Clonase™ enzyme mix	Invitrogen	Cat#11791020
Triglyceride reagent	ThermoFisher	Cat#981786
Direct-zol™ RNA MiniPrep kit	ZymoResearch	Cat#R2050
UltraScript™ cDNA synthesis Kit	PCRBiosystems	Cat#PB30.11-10
Experimental models: Organisms/strains		
Drosophila: *Canton-S*		
Drosophila: *AkhR-LexA*	This study	N/A
Drosophila: *Akh^A^*	R. Kunhlein; Galikova et al.^29^	N/A
Drosophila: *Akh^AP^*	R. Kunhlein; Galikova et al.^29^	N/A
Drosophila: *AkhR*^1^	R. Kunhlein; Galikova et al.^29^	N/A
Drosophila: *Bmm*^1^	R. Kunhlein; Gronke et al., 2007^28^	N/A
Drosophila: *w^1118^*	R. Kunhlein; Galikova et al.^29^	N/A
Drosophila: *Akhr:BD; nSyb:AD*	L. Wang; Yu et al.^10^	N/A
Drosophila: *AkhR-Gal4*	L. Wang; Yu et al.^10^	N/A
Drosophila: *Akh-Gal4*	Bloomington Drosophila Stock Center	RRID:BDSC_25684
Drosophila: *MB504B-Gal4*	Bloomington Drosophila Stock Center	RRID:BDSC_68329
Drosophila: *10xUAS-GRABDA_2m_*	Bloomington Drosophila Stock Center	RRID:BDSC_90878
Drosophila: *20xUAS-GcaMP7f*	Bloomington Drosophila Stock Center	RRID:BDSC_80906
Drosophila: *MB320C-Gal4*	Bloomington Drosophila Stock Center	RRID:BDSC_68253
Drosophila: *LexAop-CD8-GFP-2A-CD8-GFP;* *UAS-mLexA-VP16-NFAT, LexAop-rCD2-GFP*	Bloomington Drosophila Stock Center	RRID:BDSC_66542
Drosophila: *tubP-Gal80^ts^*	Bloomington Drosophila Stock Center	RRID:BDSC_7019
Drosophila: *UAS-rpr*	Bloomington Drosophila Stock Center	RRID:BDSC_5824
Drosophila: *UAS-AkhR Ri TRIP*	Bloomington Drosophila Stock Center	RRID:BDSC_29577
Drosophila: *Ppl-Gal4*	Bloomington Drosophila Stock Center	RRID:BDSC_58768
Drosophila: *Cg-Gal4*	Bloomington Drosophila Stock Center	RRID:BDSC_7011
Drosophila: *R85G01-Gal4*	Bloomington Drosophila Stock Center	RRID:BDSC_40436
Drosophila: *GMR88F08-Gal4*	Bloomington Drosophila Stock Center	RRID:BDSC_47982
Drosophila: *10xUAS-mCD8::GFP*	Bloomington Drosophila Stock Center	RRID:BDSC_32185
Drosophila: *LexAop-rCD2::RFP-p10.UAS-mCD8::GFPp10*	Bloomington Drosophila Stock Center	RRID:BDSC_67093
Drosophila: *UAS-myrGFP.QUAS-mtdTomato-3xHA; trans-*Tango	Bloomington Drosophila Stock Center	RRID:BDSC_77124
Drosophila: *26xLexAop-mCD8::GFP*	Bloomington Drosophila Stock Center	RRID:BDSC_32207
Drosophila: *UAS-TbH Ri TRIP*	Bloomington Drosophila Stock Center	RRID:BDSC_27667
Drosophila: *Gr5a-Gal4*	Bloomington Drosophila Stock Center	RRID:BDSC_57592
Drosophila: *nSyb-Gal4*	Bloomington Drosophila Stock Center	RRID:BDSC_51635
Drosophila: *UAS-Akh Ri 105063KK*	Vienna Drosophila Resource Center	RRID:VDRC_105063
Drosophila: *UAS-Akh Ri 11352 GD*	Vienna Drosophila Resource Center	RRID:VDRC_11352
Drosophila: *UAS-AkhR Ri 109300KK*	Vienna Drosophila Resource Center	RRID:VDRC_109300
Drosophila: *UAS-AkhR Ri 9546 GD*	Vienna Drosophila Resource Center	RRID:VDRC_9546
Drosophila: *UAS-Lst Ri 13891 GD*	Vienna Drosophila Center	RRID:VDRC_13891
*Drosophila: UAS-Shi^ts^*	Kitamoto et al.^33^	RRID:BDSC_44222
Drosophila: *UAS-oct-TyrR Ri 26877GD*	Vienna Drosophila Resource Center	RRID:VDRC_26877
Drosophila: *UAS-oct α2R Ri 10215GD*	Vienna Drosophila Resource Center	RRID:VDRC_10215
Drosophila: *UAS-octα1R Ri 47896GD*	Vienna Drosophila Resource Center	RRID:VDRC_47896
Drosophila: *UAS-octα2R Ri 102524KK*	Vienna Drosophila Resource Center	RRID:VDRC_102524
Drosophila: *UAS-octα3R Ri 106599KK*	Vienna Drosophila Resource Center	RRID:VDRC_106599
Drosophila: *UAS-oamα Ri2861GD*	Vienna Drosophila Resource Center	RRID:VDRC_2861
Oligonucleotides		
Primer (for AKHR-LexA): AkhR Forward: CACCAGGTAACGGTACTCCAGATCCACA Reverse: CCGGTAATCTCGCTC ATGTTGGACG	Liming Wang; Yu et al.^10^	N/A
Primer (qPCR): Akh Forward: ATCCCAAGAGCGAAGTCC Reverse: CCTGAGATTGCACGAAGC	Huang et al.^65^	N/A
Primer (qPCR): AkhR Forward: ACTGCTACGGAGCCATTT Reverse: TGTCCAGCCAGTACCACA	Huang et al.^65^	N/A
Primer (qPCR): RP49 Forward: CTTCATCCGCCACCAGTC Reverse: CGACGCACTCTGTTGTCG	Slaidina et al.^93^	N/A
Recombinant DNA		
pBpLexA::p65Uw	Addgene; Pfeiffer et al.^94^	Cat#26231
AkhR-LexA	This study	N/A
Software and algorithms		
R	R Development Core Team	http://www.R-project.org/; RRID:SCR_001905
Cytoscape	Shannon et al.^95^	https://cytoscape.org
FIJI	ImageJ	fiji.org
SimpleNeuriteTracer v4.0.3. (Fiji)	Arshadi et al.^96^	https://github.com/morphonets/SNT/
CMTKv.3.3.2.		https://www.nitrc.org/projects/cmtk/
GraphPad Prism	GraphPad	N/A
MatLab	MathWorks	N/A
Custom-made Two-Photon analysis script	This study	N/A
Blender v. 3.2.2	The Blender Community	www.blender.org
Natverse	Bates et al.^45^	https://natverse.org
Fafbseg	Bates et al.^45^	https://natverse.org/fafbseg/index.html
FlyWire	Dorkenwald et al.^44^; Schlegel et al.^97^	https://flywire.ai, RRID:SCR_019205
Codex	unpublished data	https://codex.flywire.ai
CAVEClient	unpublished data	https://github.com/seung-lab/CAVEclient
Custom connectivity analyses scripts (R based)	Available on request	N/A
Nat	Bates et al.^45^	https://natverse.org/nat/
Nat.JRCbrains	Bates et al.^45^; Bogovic et al.^98^	https://natverse.org/nat.jrcbrains/index.html
Nat.NBLAST	Bates et al.^45^; Costa et al.^46^	https://natverse.org/nat.nblast/
Adobe Creative suite (Illustrator)		N/A
VirtualFly brain		virtualflybrain.org

## Resource Availability

### Lead contact

Further information and requests for resources and reagents should be directed to and will be fulfilled by the lead contact, Scott Waddell (scott.waddell@cncb.ox.ac.uk).

### Materials availability

Plasmid and fly lines generated in this study are available upon request.

## Experimental Model Details

### Fly strains and maintenance

All *Drosophila melanogaster* strains were reared at 50% relative humidity on standard cornmeal-agar food in 12:12h light:dark cycle and were group housed. All strains were maintained at 25°C, except the temperature sensitive *UAS-Shi^ts^* flies, which were raised at 20°C. Unless otherwise specified, 3-9 day old flies were used for experiments. For all experiments both males and females were used except for TAGs measurements that only assayed males.

The following fly strains were used. The Waddell lab Canton-S stock is that labelled wildtype. *Akh*^A^, *Akh*^AP^, *AkhR*^1^, *Bmm*^1^ and *w*^1118^ were a kind gift from R. Kühnlein (Max-Planck, Göttingen, Germany). *AkhR:BD; nSyb:AD* and *AkhR-Gal4* were kindly provided by L. Wang (Zhejiang University, Hangzhou, China). *Akh-Gal4* (RRID:BDSC_25684), *MB504B-Gal4* (RRID:BDSC_68329), *10xUAS-GRAB-DA*_*2m*_ (RRID:BDSC_90878), *MB320C-Gal4* (RRID:BDSC_68253), *LexAop-CD8-GFP-2A-CD8-GFP; UAS-mLexA-VP16-NFAT, LexAop-rCD2-GFP* (RRID:BDSC_66542), *tubP-Gal80^ts^* (RRID:BDSC_7019), *UAS-rpr* (RRID:BDSC_5824), *20xUAS-GCaMP7f* (RRID:BDSC_80906), *UAS-AkhR Ri TRIP* (RRID:BDSC_29577), *ppl-Gal4* (RRID:BDSC_58768), *cg-Gal4* (RRID:BDSC_7011), *nSyb-Gal4* (RRID:BDSC_51635), *GMR88F08-Gal4* (RRID:BDSC_47982), *10xUAS-mCD8::GFP* (RRID:BDSC_32185), *UAS-myrGFP. QUAS-mtdTomato-3xHA; trans-Tango* (RRID:BDSC_77124), *26XLexAop-mCD8::GFP* (RRID:BDSC_32207), *UAS-TbH Ri TRIP* (RRID:BDSC_27667), *R85G01-Gal4* (RRID:BDSC_40436), *LexAop-rCD2::RFP-p10.UAS-mCD8::GFPp10* (RRID:BDSC_67093), *Gr5a-Gal4* (RRID:BDSC_57592) and *UAS-Shi^ts^* (RRID:BDSC_44222) were obtained from the Bloomington Drosophila Research Center, Bloomington, Indiana. *UAS-Akh Ri* 105063KK, *UAS-Akh Ri* 11352GD, *UAS-AkhR Ri* 109300KK, *UAS-AkhR Ri* 9546GD, *UAS-Lst Ri* 13891GD, *UAS-Oct-TyrR* Ri 26877GD, *UAS-Oamb Ri* 2861GD, *UAS-Oct*α*2R Ri* 10215GD, UAS-*Oct*β*1R Ri* 47896GD, UAS-*Oct*β*2R Ri* 102524KK, UAS-*Oct*β*3R Ri* 106599KK lines were acquired from Vienna Drosophila Research Center, Vienna, Austria. The *AkhR-LexA* strain was made in this study.

## Method Details

### Plasmids and Generation of transgenic lines

*AkhR* promoter sequence (-2804 to +55) was PCR amplified from genomic DNA (Nucleospin Tissue Ref 740952.50) using the Platinum™ SuperFi II PCR Master Mix (Invitrogen #12368010). The amplicon was cloned into the pENTR/D-TOPO vector (pENTR/D-TOPO cloning kit, ThermoFisher #K240020) using the following gene-specific primers: sense primer CAC CAG GTA ACG GTA CTC CAG ATC CAC A and antisense primer CCG GTA ATC TCG CTC ATG TTG GAC G. To generate *AkhR-LexA* flies, the promoter sequence was cloned into the Gateway Destination vector pBpLexA::p65Uw (addgene #26231) using the Gateway™ LR Clonase™ II enzyme mix (Invitrogen #11791020). The *AkhR*-LexA construct was then introduced into the germ line by injections into the y^1^w^67c23^; P{CaryP}attP40 fly line (BestGene).

### Behavioral experiments

For behavioral T-maze experiments, 3-9 day old mixed sex flies were used unless otherwise specified. Odors were 4-methylcyclohexanol (MCH) and 3-octanol (OCT), diluted 1:10^3^. Unless otherwise specified, all experiments were conducted at 23°C and 65-70% relative humidity. Temperature was only raised to the restrictive 30-33°C, during conditioning of the *Shi*^ts1^ experiments and for 1 to 2 days prior to conditioning when RNAi transgenes were induced using tubGal80^ts^ control.

Unless otherwise specified, flies were *ad libitum* fed and hydrated before conducting behavioral experiments. For starvation, approximately 100 mixed sex flies of 3-7 days old were kept for 20-24 h in vials containing 1% agar (water source) and a 2x4 cm strip of Whatman filter paper. For water deprivation, approximately 100 flies per vial were housed for 12 h with a 2x6 cm piece of dry sucrose-coated filter paper above drierite (Sigma Aldrich) at 25°C.^17^ The drierite was separated from the flies by a layer of cotton wool.

### Aversive olfactory conditioning with electric shock reinforcement

Aversive conditioning experiments were performed as previously described.^15,19,60^ Groups of approximately 100 flies were housed 15-24 h before conditioning in a 25 mL vial containing standard food and a 2x6 cm strip of Whatman filter paper. Flies were trained with a single cycle of aversive training. During each aversive training cycle, flies were exposed for 1 min to a first odor (CS^+^) paired with 12 electric shocks (90V) at 5 s intervals. Flies were then allowed to rest for 45s with clean air. They were then presented with a second odor (CS^-^) for 1 min without shocks. Testing was either immediately, 3 h or 24 h after training.

For CaLexA experiments, flies underwent a cycle of aversive training as described above and were tested for memory immediately after training. Flies that selected the CS^-^ arm of the T-maze were collected and housed for 4 h either in a vial containing standard food or 1% agar, before dissection.

### Appetitive olfactory conditioning with sugar reinforcement

Flies were trained with sucrose reward as previously described.^18^ Groups of approximately 100 flies were housed 15-24 h before conditioning in a 25 mL vial containing standard food and a 2x6 cm stripe of Whatman filter paper. They were then trained with one cycle of appetitive training and tested for memory immediately afterwards. During each appetitive training cycle, flies were exposed for 2 min to a first odor (CS^-^) without sugar. After 30s of clean air, flies were presented with a second odor (CS^+^) paired with sugar.

### Aversive olfactory conditioning with bitter reinforcement

Flies were trained with DEET (N,N-diethyl-meta-toluamide) as previously described.^48^ 3-7 day old mixed sex flies were starved for 20-24 h in vials containing 1% agar and a 2x4 cm strip of Whatman filter paper. Training and immediate testing were performed either at 33°C or 23°C. Flies were exposed to the CS^-^ odor with 1% agar on the filter paper for 2 min followed by 30 s of fresh air, then 2 min of CS^+^ odor with 0.4% DEET, 3M xylose and 100mM sucrose in 1% agar on filter paper. Flies were tested for their odor preference immediately after training.

For analyses of conditioning experiments, a half performance index was calculated as the number of flies in the CS^+^ arm minus the number in the CS^-^ arm, divided by the total number of flies.

$$\;half\;PI=\frac{nbCS^{+}-nbCS^{-}}{total\;nb}$$

MCH and OCT were alternately used as CS^+^ or CS^-^ and a single sample (n) represents the average of the half performance indexes from two reciprocally trained groups.

### Sensory acuity

$$PI=\frac{\;half\;PI\;mch\text{+}half\;PI\;oct\;}{2}$$

Sensory acuity tests were accomplished as described in Schwaerzel et al.,^75^ Keene et al.,^99^ and Keene et al.^100^ with modifications. For odor acuity, naïve flies were given 2 min to choose between one odor diluted 1:10^3^ (either MCH or OCT) and air bubbled through mineral oil. The avoidance index was calculated as: number of flies in the odor-arm minus the number of flies in the air-arm, divided by the total number of flies.

$$\;odor\;acuity\;=\frac{nb\;odor\;-nb\;air\;}{\;total\;nb}$$

To test electric shock avoidance or shock acuity, näve flies were given 1 min to choose between two tubes containing electric grids with only one of them connected to the power source. An avoidance index was calculated as the number of flies in the non-electrified arm divided by the total number of flies and multiplied by 100.

$$\;shock\;acuity\;=(\frac{nb\;non\;electrified\;}{\;total\;nb})\times 100$$

### Triglycerides (TAGs) measurement

5-7 day old male flies kept in standard cornmeal-agar food were used. Eight flies per replicate were frozen, homogenized in 1 mL of PBS + 0.05% Tween 20 and centrifuged 10 s at 5000 rpm. Samples were then incubated 5 min at 70°C and centrifuged 3 min at 3500rpm. 50 μL of each supernatant was transferred into a 96 well plate and homogenate absorbance was measured at 540 nm using a CLARIOstar multiplate reader. 200 μL of pre-warmed Triglyceride reagent (Thermo Scientific Ref 981786) was added to each sample and incubated 30 min at 37°C with mild shaking. Total absorbance was then measured at 540 nm. TAGs standards were made with Triolein (Sigma Aldrich T7140) and treated in the same way as the samples. TAGs levels were measured by subtracting the homogenate absorbance from the total absorbance and TAGs concentrations were then normalized by the wet weight of the flies.

### Immunostaining

Brains from 3 to 7 day old adult flies were dissected in PBS and fixed for 20 min in PBS with 4% paraformaldehyde at room temperature (RT). After 4 washes of 20 min each in 0.5% PBT (PBS + Triton X100), brains were blocked overnight at 4°C in blocking solution (PBT containing 10% normal goat serum or 10% normal donkey serum). Brains were then incubated with primary antibodies diluted in the blocking solution for 2 days at 4°C with mild rotation (45rpm), then washed 4 times for 20 min each at RT with PBT. Next, brains were incubated with secondary antibodies diluted in the blocking solution for 2 days at 4°C with mild rotation (45rpm), then washed 4 times for 20 min at RT with PBT. Stained brains were mounted on glass slides in Vectashield (Vector Labs H-1000) and imaged using a Leica TCS SP5 confocal microscope at 25x magnification (HCX PL APO 25x, 1.3CS water immersion objective, Leica). Image stacks were collected with 0.5 μm steps and processed using FIJI.^101^

The following antibodies were used: chicken anti-GFP (1/1000) (ab13970 abcam), mouse anti-nc82 (1/50) (DSHB), rat anti-HA (1/200) (3F10, Roche), rabbit anti-Dsred (1/200) (cat#632496, Clontech), rabbit anti-Tdc2^92^ (1/500) (ab128225, Abcam), mouse anti-Repo (1/200) (DSHB) and goat anti-VAchT (1/1000) (cat#ABN100, Sigma Aldrich). For the trans-Tango staining, F_0_ crosses were maintained at 18°C. F_1_ were kept at 18 °C and brains were dissected from 14-21 day old flies to allow sufficient time for trans-Tango induced RFP expression.^41^

### Fluorescence quantification of CaLexA experiments

After acquisition of confocal z-stacks with a 0.5 μm step, identical laser power and scan settings, images were analyzed using FIJI. We performed the sum-intensity 3D projections to measure total fluorescence intensity across the region of interest (PPL101 (γ1pedc) DAN axons) and subtracted the background fluorescent intensity. For a given experiment, all values were normalized to flies of fed näve condition to obtain the “GFP accumulation ratio”. n represents the PPL101(γ1pedc) neuron number.

$$\;GFP\;accumulation\;ratio\;=\frac{\;Fluorescence\;signal\;-\;Fluorescence\;background\;}{\left(average\;Fluorescence\;of\;fed\;naïve\;condition\right)}$$

Normalized trained values were divided by the normalized näve values to obtain the “GFP accumulation Trained/Naive ratio” in both fed and starved conditions. *n* represents the average of individual experimental sessions.

### *In vivo* Two-Photon dopamine imaging

Functional-imaging experiments were performed as previously described with some minor modifications.^60,63,102^ All flies were raised at 25 °C and 10-20 day old male and female flies were used. For the starved condition, flies were starved on 1% agar for 15 to 19 h before recording. Flies were immobilized on ice and mounted in a custom-made chamber allowing free movement of the legs and antennae. The head capsule was opened under carbogenated buffer solution (95% O_2_, 5% CO_2_) (103 mM NaCl, 3 mM KCl, 5 mM N-Tris, 10 mM trehalose, 10 mM glucose, 7 mM sucrose, 26 mM NaHCO_3_, 1 mM NaH_2_PO_4_, 1.5 mM CaCl_2_, 4 mM MgCl_2_, osmolarity 275 mOsm, pH 7.3). Individual flies in the recording chamber were placed under a two-photon microscope and an electric shock grid was positioned in contact with the fly’s legs.

One hemisphere of the brain was randomly selected to image the GRAB_DA2m_ fluorescence signal from the γ1pedc mushroom body compartment. Fluorescence was excited using ~140 fs pulses, 80 MHz repetition rate, centred on 910 nm generated by a Ti-Sapphire laser (Chameleon Ultra II, Coherent). Flies were exposed to electric shocks and images of 256x256 pixels were acquired at 5.92 Hz using a Two-Photon microscope (Scientifica) with a 40x, 0.8NA water immersion objective, controlled by ScanImage 3.8 software.

Six 1.5 s 90V electric shocks were provided for 1 min (0.1Hz). A custom-made device was used to record the current passing through the fly, so that shock responses could be selected from those when the current was registered. To image electric shock responses, each fly was recorded 30 s before the onset of the 1 min sequence of electric shocks. As a quality control, each fly was presented with an odor for 1 min, 45 s after the end of the shock presentation. Any flies not showing an odor response in the γ1pedc compartment were excluded from the analyses.

To analyse the data, recorded images were manually segmented using FIJI. Fly movements were small enough such that images did not require registration. For each recording, one region of interest (ROI) was drawn around the area expressing GRAB_DA2m_ to generate the summed fluorescence at each frame. A second ROI of the same size was chosen in the background where no changes occur during the whole recording. Subsequent analyses were made using custom-made Excel macros and MatLab scripts (Method S1). The GRAB_DA2m_ fluorescence F was then calculated by subtracting the background. The baseline fluorescence F_0_ was defined for each shock response as the mean fluorescence F between 1.5 s and 0.5 s before each electric shock onset. For graphs relating F_0_ values each *n* represents the averaged F_0_ per fly. Variations of the GRAB_DA2m_ fluorescent were calculated from the baseline as follows: ΔF/F_0_= (F-F_0_)/F_0_.

Each *n* corresponds to the average of electric shock responses per fly as follows:

- the peak ΔF/F_0_ corresponds to the highest ΔF/F_0_ value over the duration of the shock for each fly.
- the AUC corresponds to the area under the curve of the averaged shock response for each fly, over 1.5 s or 5 s after shock onset.

To analyse odor responses, the baseline fluorescence F_0_ was defined as the mean fluorescence F between 11.5 s and 1.5 s before each odor onset. Each *n* represents the F_0_ per fly. Variations of the fluorescence were calculated as described for shock responses. Since odor responses decayed quickly back to baseline, the peak ΔF/F_0_ corresponds to the highest ΔF/F_0_ value over the first 5 s after odor onset, and AUC corresponds to the area under the curve of the odor response for 5 s after odor onset. Each n corresponds to one fly.

### *In vivo* Two-Photon calcium imaging – synthetic AKH bath application

Functional-imaging experiments were performed as described above with some minor modifications. All flies were raised at 25 °C and 2-6 day old male and female flies were used. Flies were immobilized on ice and mounted upside down in a custom-made chamber allowing to dissect them under the proboscis in order to visualize AKHR neurons.

For acute synthetic AKH peptide application, we used a perfusion pump system (14-284-201, Fisher Scientific) to continuously deliver buffer solution at a rate of approximately 0.043mL/s. Synthetic AKH (1 nM) was applied with and without the presence of 100 nM tetrodotoxin (TTX), to block voltage-gated sodium channels and propagation of action potentials that could result in indirect excitation. Owing to perfusion tubing length and dead volume, the perfusion switch took approximately 600 frames (100s) to reach the brain.

The synthetic AKH peptide (sequence: Glp-LTFSPDW-NH2) was made by Sigma Aldrich.

To verify that AKHR neurons of flies with the *AkhR^1^* mutant background were still able to respond we followed application of 1nM AKH with delivery of 30 mM KCl.

To analyse the data, recorded images were manually segmented using FIJI. Fly movements were small enough such that images did not require registration. For each recording, one region of interest (ROI) was drawn around the area expressing GCaMP7f to generate the summed fluorescence at each frame. A second ROI of the same size was chosen in the background where no changes occur during the whole recording. The GCaMP7f fluorescence F was then calculated by subtracting the background.

The baseline fluorescence F_0_ was defined as the mean fluorescence F 200 frames before AKH application (600 frames for Figure S3L). Variations of the GCaMP7f fluorescent were calculated from the baseline as follows: ΔF/F_0_= (F-F_0_)/F_0_. AUC corresponds to the area under the curve of the pre-, during and post- response for 200 frames (600 frames for Figure S3L) after AKH application. Each n corresponds to one fly.

### Connectomics analyses

#### Data origin and handling

Neuron skeletons, synapses, mesh data, and connectivity information originating in the FAFB (Full adult female brain) electron microscopy dataset^43^ were accessed via FlyWire (flywire.ai/^44,97^, the connectome data explorer - Codex (codex.flywire.ai/), supported by BRAIN Initiative grant MH117815, MH129268 and U24 NS126935 to Murthy and Seung), Connectome Annotation Versioning Engine (CAVEclient, https://github.com/seung-lab/CAVEclient) and natverse (https://natverse.org, v0.24,^45^ Development of natverse including the fafbseg package has been supported by the NIH BRAIN Initiative (1RF1MH120679-01), NSF/MRC Neuronex2 (NSF 2014862/MC_EX_MR/T046279/1) and core funding from the Medical Research Council (MC_U105188491). Synapse data was obtained via natverse (fafbseg::flywire_partner_summary; V0.12.0; timestamp - 2023-07-10 15:00:00 UTC) and automated synapse and neurotransmitter predictions^51,103^ were accessed via the CAVEclient. Synapses with a cleft threshold >50 were included in our analysis.^104^ Data analyses and morphological clustering was carried out with custom scripts based on natverse functions in R (V 4.2.2) (available upon request). 3D mesh data was obtained via cloudvolume (https://github.com/seung-lab/cloud-volume/) from FlyWire’s Neuroglancer data (https://github.com/google/neuroglancer, flywire-daf.com/segmentation/1.0/fly_v31) with python based custom scripts applying navis (v.1.2.1, https://github.com/navis-org/navis) functionalities. The registered brain mesh of the FAFB brain (FAFB14) was obtained from navis-flybrains (https://pypi.org/project/flybrains/).

### Identification of AKHR neurons

AKHR neurons were identified based on morphological features upon inspection of raw EM data and 3D representations via neuroglancer in FlyWire (https://ngl.flywire.ai). The localization of uncharacteristically shaped, large nuclei outside the surface glial sheath in the proximal part of the pharyngeal nerve and axonal projections in the prow region were key morphological identifiers.

### Tracing AkhR-Split10xUAS-mCD8::GFP neurons

Registration: Fiji cmtk Registration (https://github.com/jefferis/fiji-cmtk-gui) and transformation with parameters -X 26 -C 8 -G 80 -R 4 -A ‘–accuracy 0.4’ -W ‘–accuracy 0.4, into JRC2018U template brain space (template brain: nat.flybrains, https://natverse.org/nat.flybrains/).

Tracing: SNT v4.2.1. tracing of the main branches of a likely AKHR neuron from the soma.

### AKHR>GFP signal vs. AKHR flywire meshes vs AKHR>GFP trace morphology comparison

Dotprops of the GFP channel obtained from a Akhr:BD; nSyb:AD, 10xUAS-mCD8::GFP, brain, that was previously registered and transformed into JRC2018U space (see above) were generated with navis.read_nrrd(output=’dotprops’, k=6, threshold=12).

Meshes of AKHR_neurons from flywire were mirrored and transformed with navis.xfrom_brain() and navis.mirror_brain(). Comparative images were generated with navis.plot3d().

### Top10 AKHR neuron matches and NBLAST comparison

All by all NBLAST data to determine 10 most similar neurons to each putative flywire AKHR skeleton were obtained from Schlegel et al.^97^ A one by one NBLAST comparison (mean of normalised forward and reverse NBLAST scores) was then performed between each AKHR skeleton and its 10 most similar neurons. The GFP trace (see above) was compared to each of its 10 most similar skeletons from this collection as well.

### Identification of ascending neurons downstream of AKHR neurons that connect to DANs

AKHR downstream neurons were identified via fafbseg:: flywire_partner_summary. The resulting neurons were filtered for ascending neurons identified by morphology and synapse locations (inputs predominantly in the SEZ and outputs predominantly in the superior brain neuropils close to DAN dendrites). Only PAM and PPL1 DANs that were downstream of ascending neurons were included in analyses. No minimum connectivity threshold was applied.

Neuronal backbones were obtained via manual tracing of trans-Tango labelled neurons from registered and transformed brains (with cmtk (via fiji wrappers) and xform_brain (natverse) as described in (github.com/jefferislab/BridgingRegistrations) with the Simple Neurite Tracer (SNT, v4.0.3.Fiji https://github.com/morphonets/SNT/ ^96^). The resulting traces were compared to SEZ ascending neurons that we previously reconstructed in FAFB via Catmaid^42^ and thereby identified as SEZON01 neurons. SEZON01 neuron skeletons were imported into FlyWire previously as seeds for reconstructions and can be found with V630 root ids (7205759406 39485648,720575940629663596,720575940618926757,720575940626519704,720575940630620687,720575940638597283,720 575940640464373,720575940645859972,720575940628046635,720575940623301256) in Codex and ngl.flywire. 7 of those 10 SEZON01 neurons are downstream of AKHR neurons.

### Clustering of AKHR-downstream ascending neurons and DANs

For morphology clustering neurons were simplified using nat::prune_twigs; twig_length = 5000 (V1.10.4) and those in the left hemisphere mirrored to the right using nat.jrcbrains::mirror_fafb (V0.3.2).^98^ The neurons were then converted into dotprops. Firstly, neurons were converted from nanometers to micrometers(neuron/1000) then using nat::dotprops(resample=10, k=5). Next, an NBLAST was performed to obtain an all-by-all matrix.^46^ Clustering was performed using nat.nblast::nhclust (V.1.6.5) with ward.D2 as the clustering criterion. Clusters were initially split into 36 clusters. One cluster was further subdivided after expert inspection to yield the final 38 clusters (Video S2). PAM and PPL1 DANs were clustered based on their hemibrain type.^47^

### Connectivity representation

Edge lists were created based on connectivity (see above) between single AKHR-, ascending-, and dopaminergic neurons or clusters of these and displayed with cytoscape (v.3.9.1, ^95^). Edge weight (3 - 30 a.u.) was pass-through mapped onto the synapse number per connection (1 to 233; minimum at 1 synapse, maximum at 233 synapses). Edge transparency was continuously mapped between the pixel values of 23 and 233 onto the synapse number per connection. Edges were bundled with default parameters (n.o.h. = 3, s.c. = 0.0003, c.t. = 0.3, m.i. = 500).

### 3D representations of neurons and brain meshes

Blender (v.3.2.2; blender foundation, https://www.blender.org/) was used for composition and mesh data was programmatically handled via the inbuilt python console (v.3.10.2) with custom python scripts employing navis (v.1.2.1) functionalities (github.com/navis-org/navis). Full resolution neuron and FAFB14 brain meshes were obtained and handled as described above. Additional information on neuron identity and community labels was obtained via Codex which serves data from the FlyWire connectome.

### RNA extraction and Quantitative real time PCR

Each sample (20 flies) have been collected in liquid nitrogen and homogenized in 500μL of TRIzol. To extract mRNA we used the Direct-zol™ RNA MiniPrep kit (Zymo Research, cat. #R2050). cDNA have been synthetized from RNA samples using the UltraScript™ cDNA synthesis Kit (PCRBiosystems cat. #PB30.11-10).

qRT-PCR have then been performed using the SYBR Green I Master Mix. n represents biological replicates.
Akh primers: Forward ATCCCAAGAGCGAAGTCC and Reverse CCTGAGATTGCACGAAGC
AkhR primers: Forward ACTGCTACGGAGCCATTT and Reverse TGTCCAGCCAGTACCACA
RP49^93^ primers: Forward CTTCATCCGCCACCAGTC and Reverse CGACGCACTCTGTTGTCG

## Quantification and Statistical Analyses

Statistical analyses were performed in GraphPad Prism.

All statistical tests used and p values are listed (Table S2).

All behavioral and qRT-PCR data were analyzed with an unpaired t-test or a one-way ANOVA followed by a posthoc Tukey’s multiple comparisons test for Gaussian distributed data. Otherwise, for non-Gaussian distributed data, either a Mann-Whitney or a Kruskal and Wallis test followed by a Dunn’s multiple comparisons test were performed. To determine the Gaussian distribution of data, a Shapiro-Wilk test was done if n<50 whereas a Kolmogorov-Smirnov test was executed if n>50. No statistical methods were used to predetermine sample size.

For Two-photon imaging of dopamine experiments normality was tested using Shapiro-Wilk test normality test. Depending on the results of the normality test, either an unpaired t-tests or Mann-Whitney tests were used. For Two-photon imaging of calcium experiments normality was tested using Shapiro-Wilk test. As the samples are paired, if the distribution was normal, a RM one way ANOVA test followed by Tukey’s multiple comparisons test was performed, otherwise a Friedman test followed by Dunn’s multiple comparisons test were done.

For behavioral experiments *n* represents the average of the half performance indexes from two reciprocally trained groups (approximately 200 flies per *n*). For *in vivo* two-photon dopamine imaging *n* represents a single fly. For fluorescence quantification of CaLexA experiments *n* represents either the PPL101(γ1pedc)/AKHR neuron numbers (Figures 1B and S3M) or the average of individual experimental sessions (Figure 1B″). For TAGs measurements *n* represents eight adult flies per replicate. For qRT-PCR data, *n* represents 20 adult flies per biological replicate

## Supplementary Material

Supplemental information can be found online at https://doi.org/10.1016/j.neuron.2024.04.035.

Fig S1-S7

Table S1

Table S2

Video S1

Video S2

## Data Availability

All data reported in this paper will be shared by the lead contact upon request. All original code is available in this paper’s supplemental information. Any additional information required to reanalyze the data reported in this paper is available from the lead contact upon request.

## Highlights

- AKH, the fly glucagon, sets baseline and hunger-enhanced aversive learning levels
- AKH acts through four neurons in the SEZ, whose somata have access to the hemolymph
- AKHR neuron-released octopamine modulates inputs to reinforcing dopaminergic neurons
- Enhanced input elevates learning-relevant dopamine release and memory performance

## In brief

Meschi et al. discover a detailed neuronal mechanism through which a hormonal signal of nutrient deficit modulates aversive learning. Hormone-responsive neurons in the base of the fly brain enhance the gain of ascending input pathways that transduce punishing shock and bitter taste to dopaminergic neurons that write aversive memories.
