# Supplementary material for: Compensatory enhancement of input maintains aversive dopaminergic reinforcement in hungry Drosophila
Source: Neuron. Author manuscript; Available in PMC 2025 Dec 24. (PMC7618526; doi:10.1016/j.neuron.2024.04.035)
Supplement: Fig S1-S7 [file EMS211459-supplement-Fig_S1_S7.pdf]

Neuron, Volume *112*

Supplemental information

Compensatory enhancement of input maintains aversive dopaminergic reinforcement in hungry *Drosophila*

Eleonora Meschi, Lucille Duquenoy, Nils Otto, Georgia Dempsey, and Scott Waddell

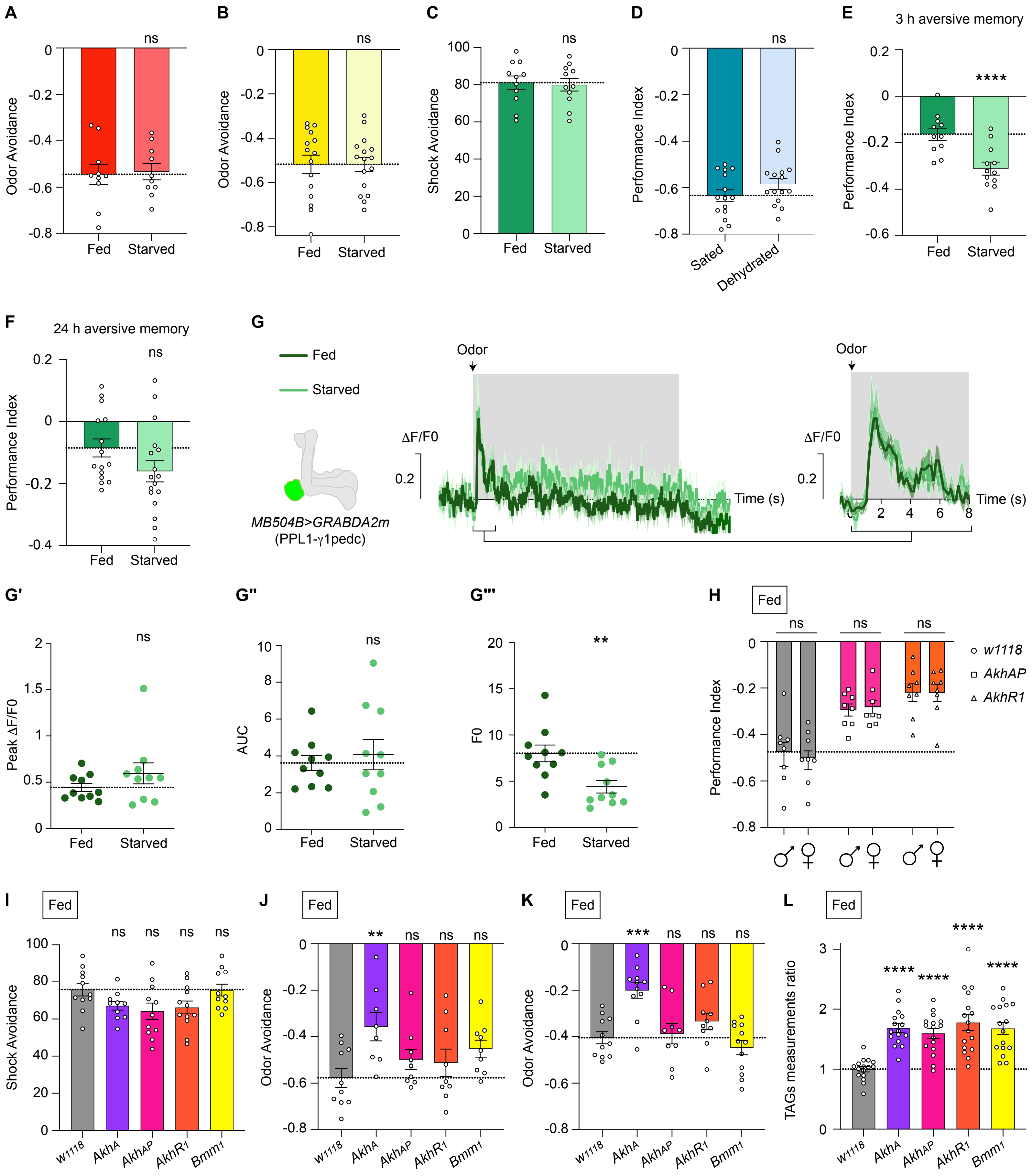

Figure S1, related to Figure 1.
Starvation enhances aversive learning through AKH.
A and B. Chronic starvation does not alter acuity of the odors used in training. **A.** MCH avoidance in naïve fed and starved wild-type flies, n=10. **B.** OCT avoidance in naïve fed and starved wild-type flies, n≥15. **C.** Chronic starvation does not impair shock avoidance. Shock avoidance in naïve fed and starved wild-type flies, n=11. **D.** Water deprivation does not alter aversive memory. Immediate aversive memory in water sated and 12 h dehydrated adult wild-type flies, n=15. **E.** Chronic starvation before training enhances aversive memory measured 3 h later, n≥11. **F.** 24 h aversive long-term memory (Fasting LTM) was not statistically enhanced in chronically (24 h) starved flies, n≥15. **G.** Chronic starvation did not enhance odor-evoked responses of PPL1-γ1pedc DANs. Mean ΔF/F0 dopamine

transients measured *in vivo* from PPL1-γ1pedc DANs of fed and starved flies, n=10. *UAS-GRAB$_{DA2m}$* driven by *MB504B-split-GAL4*. Grey rectangle represents 1 min odor presentation, n=10. **G'.** Quantification of peak $\Delta F/F_0$ DA transients measured in G. **G''.** Quantification of mean area under curve calculated during 5 s odor exposure in G, n=10. **G'''.** Quantification of baseline GRAB$_{DA2m}$ values, F_0, in fed and starved flies in G, n=10. **H.** Immediate aversive memory performance of fed male and female *w^{1118}*, *Akh^{AP}*, and *AkhR^{1}* mutant flies, n=8. **I.** Naïve shock avoidance of fed *Akh^{A}*, *Akh^{AP}*, *AkhR^{1}* and *Bmm^{1}* mutant flies, n≥10. **J.** OCT avoidance of fed *Akh^{A}*, *Akh^{AP}*, *AkhR^{1}* and *Bmm^{1}* mutant flies, n≥8. **K.** MCH avoidance of fed *Akh^{A}*, *Akh^{AP}*, *AkhR^{1}* and *Bmm^{1}* mutant flies, n≥9. **L.** TAGs measurements in *w^{1118}*, *Akh^{A}*, *Akh^{AP}*, *AkhR^{1}* and *Bmm^{1}* mutant flies, n=16.

Data presented as mean ± SEM. Individual data points of behavioral graphs represent independent groups of approximately 200 flies. Asterisks denote significant difference: **p<0.01, ***p<0.001, ****p<0.0001.

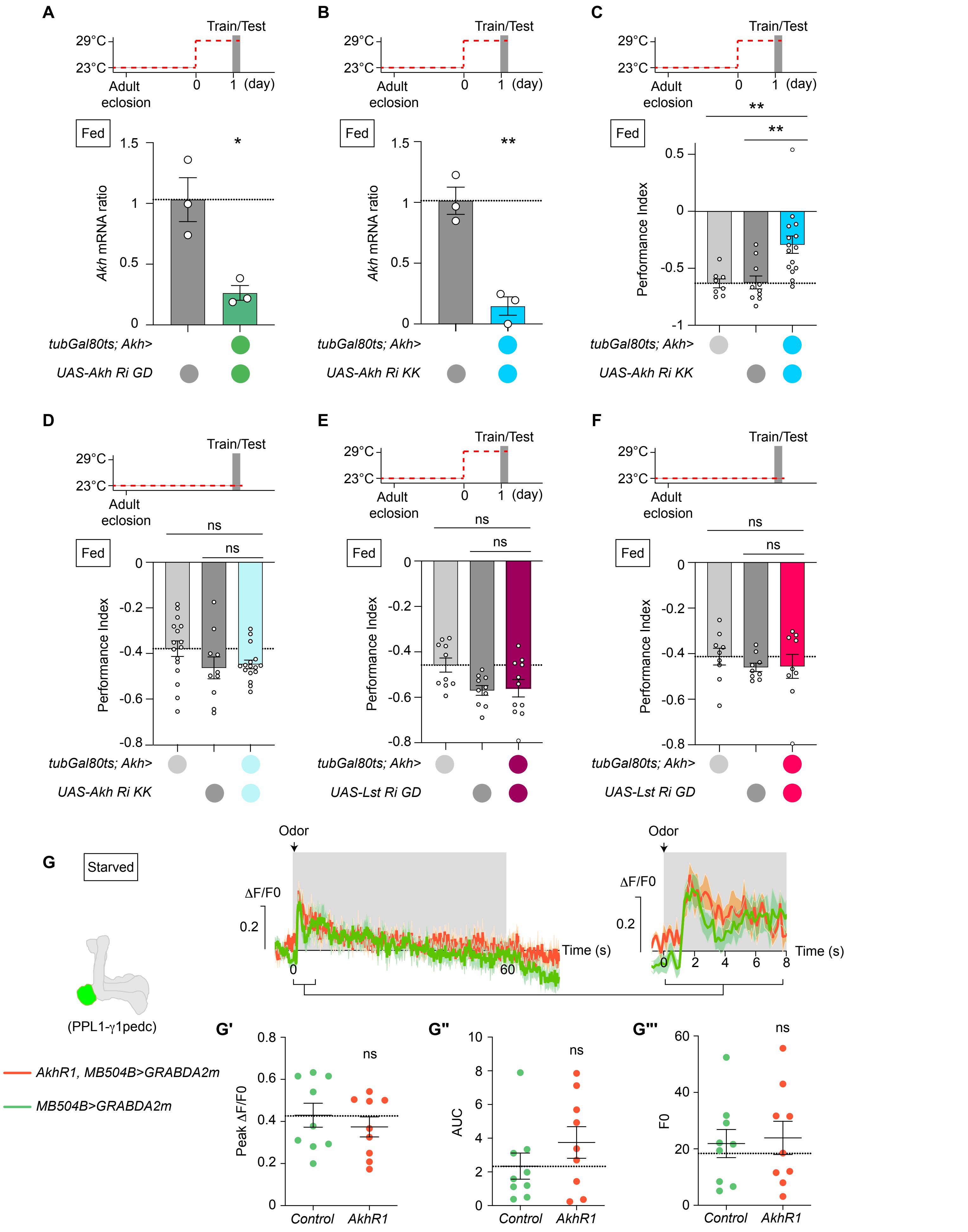

Figure S2, related to Figure 2.

AKH, but not Limostatin, is required during adulthood for normal aversive learning.

A. *Akh* mRNA levels in whole flies were quantified following adult-restricted *Akh* knockdown in the CC (*tubGal80ts; Akh>UAS-Akh Ri GD* flies), n=3. Top: temperature shifting protocol. **B.** *Akh* mRNA levels in whole flies were quantified upon adult-restricted *Akh* silencing

in the CC (*tubGal80ts; Akh> UAS-Akh Ri KK* flies), n=3. Top: temperature shifting protocol. **C.** *Akh* knockdown in the adult CC impairs immediate aversive memory (*tubGal80ts; Akh>UAS-Akh Ri KK* flies), n≥8. Top: temperature shifting protocol for RNAi induction in adulthood one day before training and testing. **D.** Permissive temperature control for *Akh* RNAi knockdown during adulthood, shown in C, n ≥7. Top: temperature protocol. **E.** Adult-restricted *Lst* knockdown in the CC did not alter immediate aversive memory (*tubGal80ts; Akh>UAS-Lst Ri GD* flies), n≥10. Top: temperature shifting protocol. **F.** Permissive temperature control for *Lst* RNAi knockdown during adulthood, shown in E, n=9. Top: temperature protocol. **G.** Mean odor-evoked $\Delta F/F_0$ dopamine transients measured *in vivo* from PPL1-γ 1pedc DANs of hungry wild type and *AkhR1* mutants flies, n=9. *UAS-GRAB*$_{DA2m}$ driven by *MB504B-split-GAL4* in wild-type and *AkhR1* mutant backgrounds. Grey rectangle marks 1 min odor presentation. **G'.** Quantification of peak $\Delta F/F_0$ dopamine transients measured in G. **G''.** Quantification of mean area under curve calculated during 5 s odor presentation of recordings in G. **G'''.** Quantification of baseline GRAB$_{DA2m}$ values, F_0, in wild-type and *AkhR1* flies in G.

Data presented as mean ± SEM. Individual data points of quantitative mRNA graphs displayed as dots represent independent groups of 20 flies. Individual data points of behavioral graphs displayed as dots represent independent groups of approximately 200 flies. Asterisks denote significant difference: *p<0.05, **p<0.01.

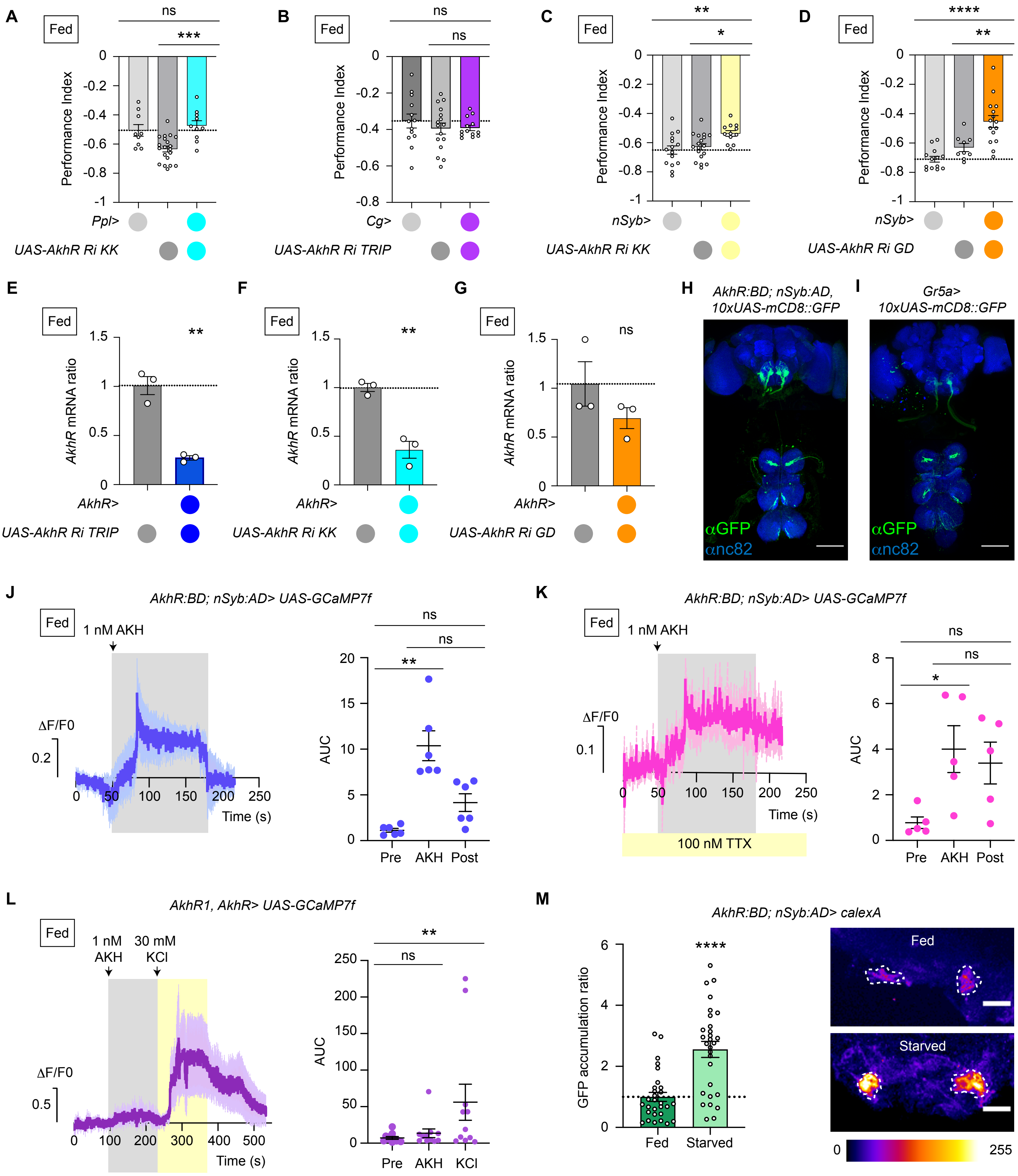

Figure S3, related to Figure 3.

Neuronal AkhR is necessary for aversive learning.

A. RNAi knockdown of *AkhR* specifically in the fat body did not alter aversive learning (*ppl>AkhR Ri KK* flies), n≥9. **B.** RNAi knockdown of *AkhR* specifically in the fat body did not alter aversive learning (*cg>AkhR Ri TRIP* flies), n≥13. **C.** Pan-neuronal RNAi knockdown of *AkhR* impaired aversive learning (*nSyb>AkhR Ri KK* flies), n≥12. **D.** Pan-neuronal RNAi knockdown of *AkhR* impaired aversive learning (*nSyb>AkhR Ri GD* flies), n≥9. **E.** *AkhR* mRNA levels in whole flies quantified upon *AkhR* knockdown in AKHR-expressing

tissues (*AkhR> UAS-AkhR Ri TRIP* flies), n=3. **F.** *AkhR* mRNA levels in whole flies quantified upon *AkhR* silencing in AKHR-expressing tissues (*AkhR> UAS-AkhR Ri KK* flies), n=3. **G.** *AkhR* mRNA levels in whole flies quantified upon *AkhR* downregulation in AKHR-expressing tissues (*AkhR> UAS-Akhr Ri GD* flies), n=3. **H.** *AkhR-split-GAL4* drives *UAS-mCD8::GFP* (green) in two AKHR neurons per hemisphere of the subesophageal zone (SEZ), in Gr5a-expressing GRNs and in some neurons in the ventral nerve cord. Neuropil generally stained with anti-nc82 (blue). Scale bar 50 µm. **I.** *Gr5a-GAL4* drives *UAS-mCD8::GFP* (green) in Gr5a-expressing GRNs and in some neurons in the ventral nerve cord. Neuropil is stained with anti-nc82 (blue). Scale bar 50 µm. **J.** Left: Mean AKH-evoked $\Delta F/F_0$ calcium transients measured *in vivo* from AKHR neurons, n=6. *UAS-GCaMP7f* driven by *AKHR-split-GAL4*. Grey rectangle marks 2 min 1nM synthetic AKH bath application. Right: Quantification of mean area under curve calculated 30 s prior, during and post AKH bath application of recordings shown on left. **K.** Left: Mean AKH-evoked $\Delta F/F_0$ calcium transients measured *in vivo* from AKHR neurons in the presence of tetrodotoxin (TTX), n=5. *UAS-GCamp7f* driven by *AKHR-split-GAL4*. Grey rectangle marks 2 min bath application of 1nM AKH. Yellow rectangle marks 100nM TTX bath application. Right: Quantification of mean area under curve calculated 30 s prior, during and post AKH bath application of recordings shown on left. **L.** Mean AKH-evoked and KCl-evoked $\Delta F/F_0$ calcium transients measured *in vivo* from AKHR neurons of *AkhR1* mutant flies, n=11. *UAS-GCaMP7f* driven by *AKHR-GAL4* in *AkhR1* mutant background. Grey rectangle marks 2 min bath application of 1 nM AKH. Yellow rectangle marks 2 min application of 30 mM KCl. Right: Quantification of mean area under curve calculated 100 s prior, during AKH and during KCl bath applications of recordings shown on left. **M.** Left: Quantification of CaLexA recorded AKHR neuron activity in fed and starved flies, n=29. Each data point represents value from a single AKHR neuron. Right: Examples of CaLexA recorded AKHR neuronal activity in fed and starved flies. *UAS-CaLexA* driven with *AKHR-split-GAL4*. Scale bars 20 µm.

Data presented as mean ± SEM. Individual data points of quantitative mRNA graphs displayed as dots represent independent groups of 20 flies. Individual data points of behavioral graphs displayed as dots represent independent groups of approximately 200 flies. Asterisks denote significant difference: *p<0.05, **p<0.01, ***p<0.001, ****p<0.0001.

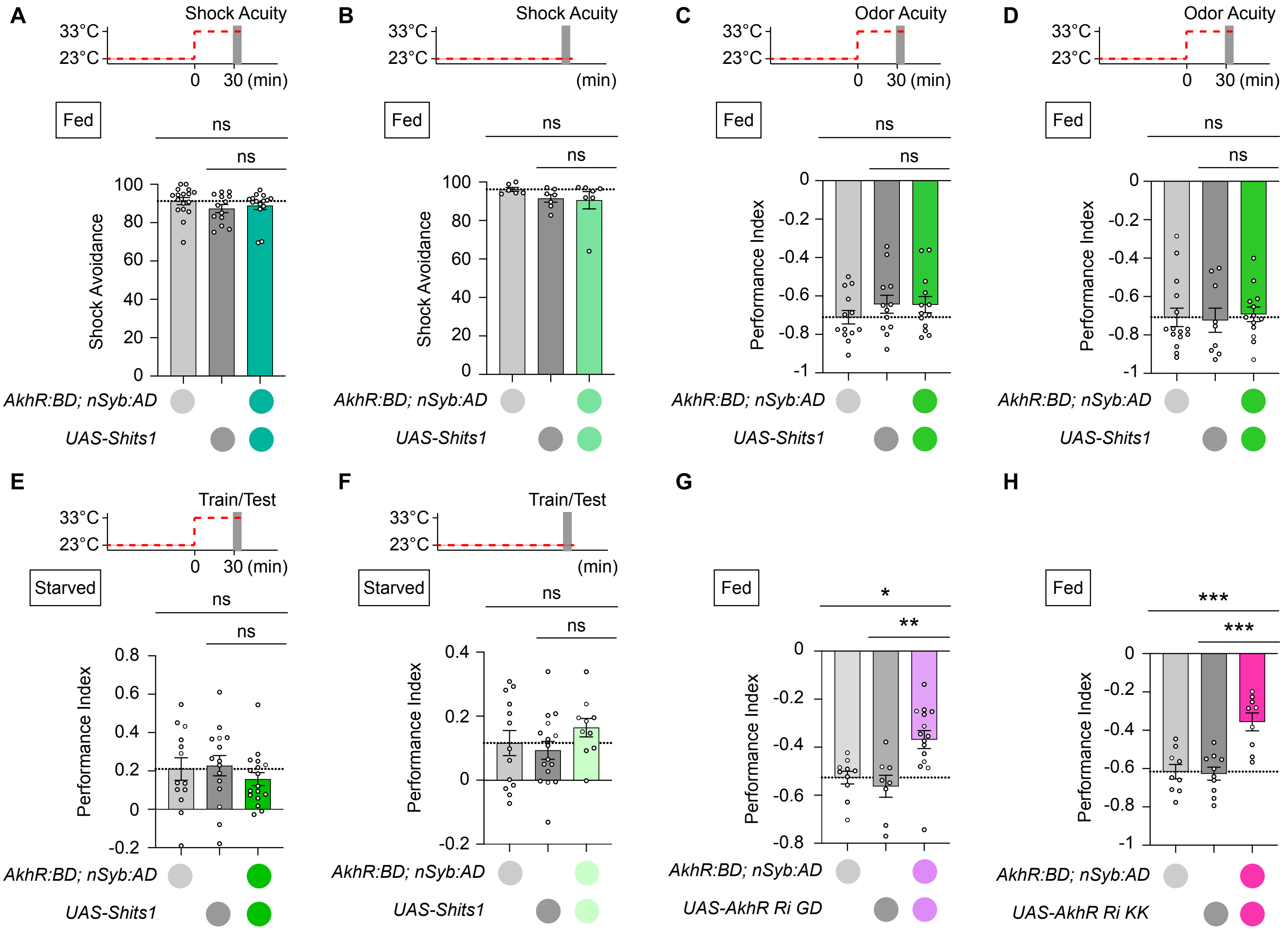

Figure S4, related to Figure 3.

AkhR is required in specific SEZ neurons for aversive learning.

A and B. Top: temperature shifting protocol. **A.** Blocking output from AKHR neurons 30 min before testing does not alter naïve shock avoidance (*AkhR::BD; nSyb::AD>UAS-Shits1* flies), n≥13. **B.** Permissive temperature control for shock avoidance experiment in A, n≥ 6. **C and D.** Blocking output from AKHR neurons 30 min before testing does not alter naïve odor avoidance (*AkhR::BD; nSyb::AD>UAS-Shits1* flies) Top: temperature shifting protocols. **C.** Naïve avoidance of MCH, n≥12. **D.** Naïve avoidance of OCT, n≥9. **E.** Blocking AKHR neuron output for 30 min before and during training and testing does not alter sugar-reinforced immediate appetitive memory (*AkhR::BD; nSyb::AD >UAS-Shits1* flies), n≥13. Top: temperature shifting protocol. **F.** Permissive temperature control for Shits1 block of AKHR neurons during appetitive memory, shown in E, n≥10. Top: temperature protocol. **G.** *AkhR* knockdown in AKHR neurons impairs immediate aversive memory (*AkhR::BD; nSyb::AD>AkhR Ri GD* flies), n≥8. **H.** *AkhR* knockdown in AKHR neurons impairs immediate aversive memory (*AkhR::BD; nSyb::AD>AkhR Ri KK* flies), n≥9.

Data presented as mean ± SEM. Individual data points displayed as dots represent independent groups of approximately 200 flies. Asterisks denote significant difference: *p<0.05, **p<0.01, ***p<0.001.

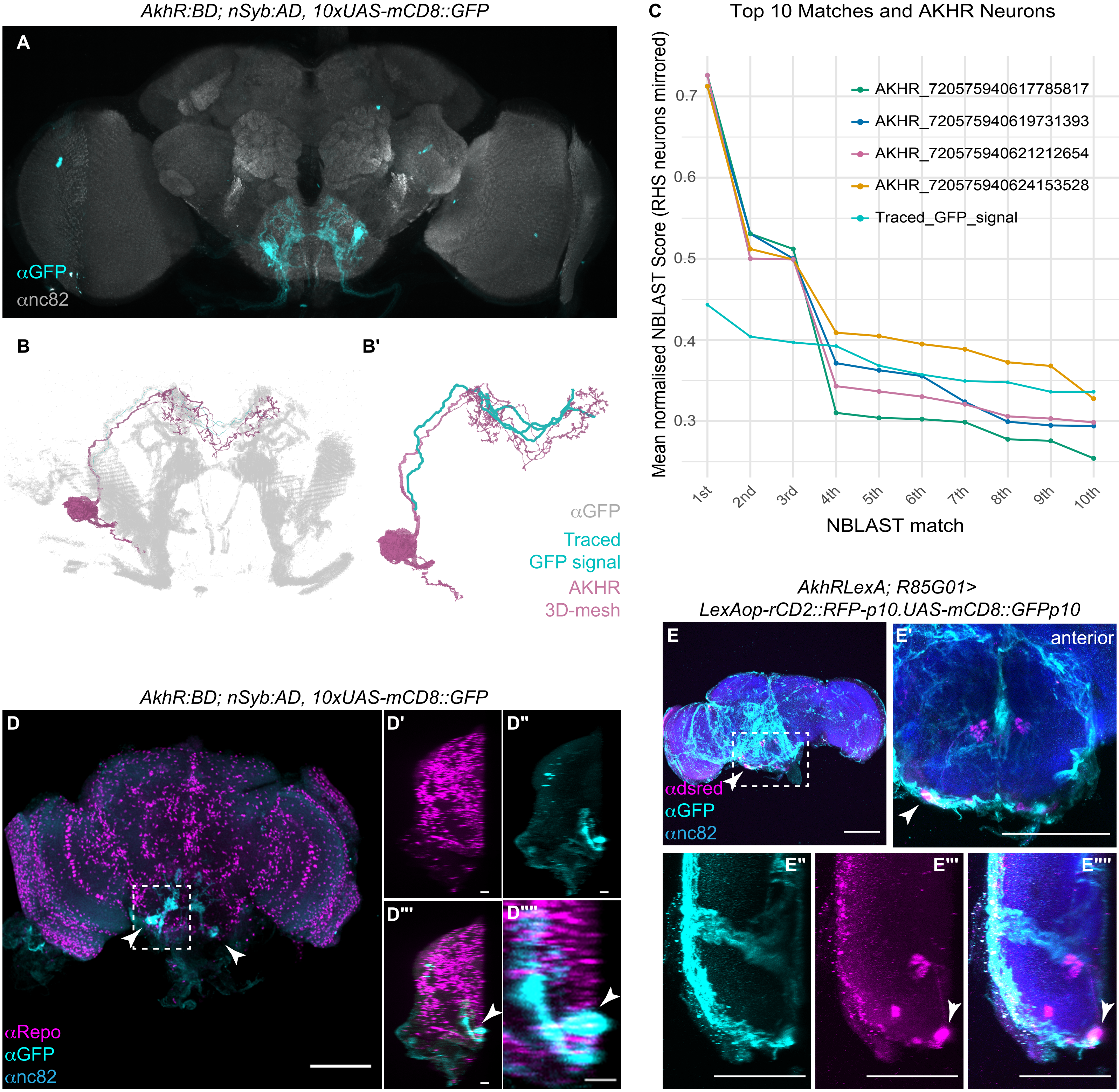

Figure S5, related to Figure 4.

AKHR neuron confirmation by NBLAST and localization of somata outside the perineurial glial sheath.

A. An *AkhR:BD; nSyb:AD, 10xUAS-mCD8::GFP* (cyan) and anti-nc82 (gray) labelled brain was registered to the JRC2018U template brain space and transformed. **B.** The *AkhR:BD; nSyb:AD, 10xUAS-mCD8::GFP* signal (gray) was converted to dotprops and is shown with a Flywire AKHR 3D-mesh transformed to JRC2018U space. Soma positions match and part of the rest of the signal clearly overlaps. **B'.** Within the *AkhR:BD; nSyb:AD, 10xUAS-mCD8::GFP* signal, the most likely AKHR neurites and the soma tract were traced (cyan) and overlayed with the Flywire AKHR 3D-mesh. The GFP signal trace overlaps with the Flywire 3D mesh. **C.** AKHR identity was confirmed and distinguished from the most similar neurons in Flywire with NBLAST. The 10 best matches to each of the 4 identified AKHR neurons were selected by highest NBLAST value 92. The best match to each AKHR neuron was its ipsilateral partner, and the second and third best matches were the contralateral AKHR neurons. Matches 4-10 for each AKHR neuron are visibly different neurons (Video S1). In addition, the GFP trace obtained in B' was compared to all 4 Flywire AKHR skeletons and their most similar neurons' skeletons and again the highest similarity was found to be the ipsi- and contralateral AKHR neurons. **D.** *AkhR-Split-GAL4* driven *10xUAS-mCD8::GFP* (cyan) shows that AKHR neuron somata (white, arrowhead) localize to the periphery compared to glial nuclei stained with anti-Repo (magenta). Scale bar 100 µm. **D'-D'''.** Lateral views of the SEZ showing (**D'**) glial nuclei (magenta), (**D''**) AKHR neurons (cyan) and (**D'''**) the merge. Scale bar 10 µm. **D''''.** Magnified lateral view of the merge. Brain neuropil visualized with

anti-nc82 (blue). Scale bar 10 µm. **E.** AKHR neuron (magenta) somata (arrow heads) are located outside the perineurial glial sheath (cyan) (*AkhR-LexA; R85G01> LexAop-rCD2::RFP-p10, UAS-mCD8::GFP*). **E'.** Magnified views of the anterior confocal projection of the SEZ showing the merge, **E''-E''''.** Lateral views of the perineurial glial sheath (cyan) (**E''**), AKHR neurons (magenta) (**E'''**) and merge (**E''''**). Brain neuropil generally stained with anti-nc82 (blue). Scale bar 100 µm.

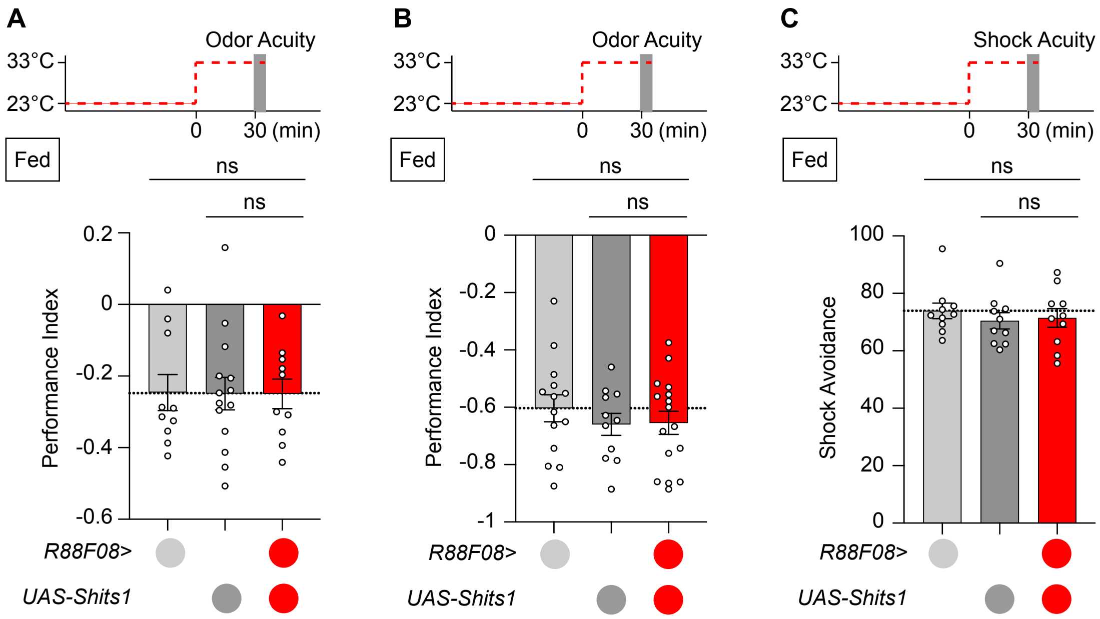

Figure S6, related to Figure 5.
SEZON01 neuron block does not impair olfactory or shock acuity.
A and B. Blocking R88F08 neuron output with *UAS-Shits1* for 30 min before and during testing does not alter olfactory acuity. **A.** MCH, n≥10. **B.** OCT, n≥11. **C.** Blocking R88F08 neuron output with *UAS-Shits1* for 30 min before and during testing does not impair naïve shock avoidance, n=10. Top in all panels: temperature shifting protocol.
Data presented as mean ± SEM. Individual data points displayed as dots represent independent groups of approximately 200 flies.

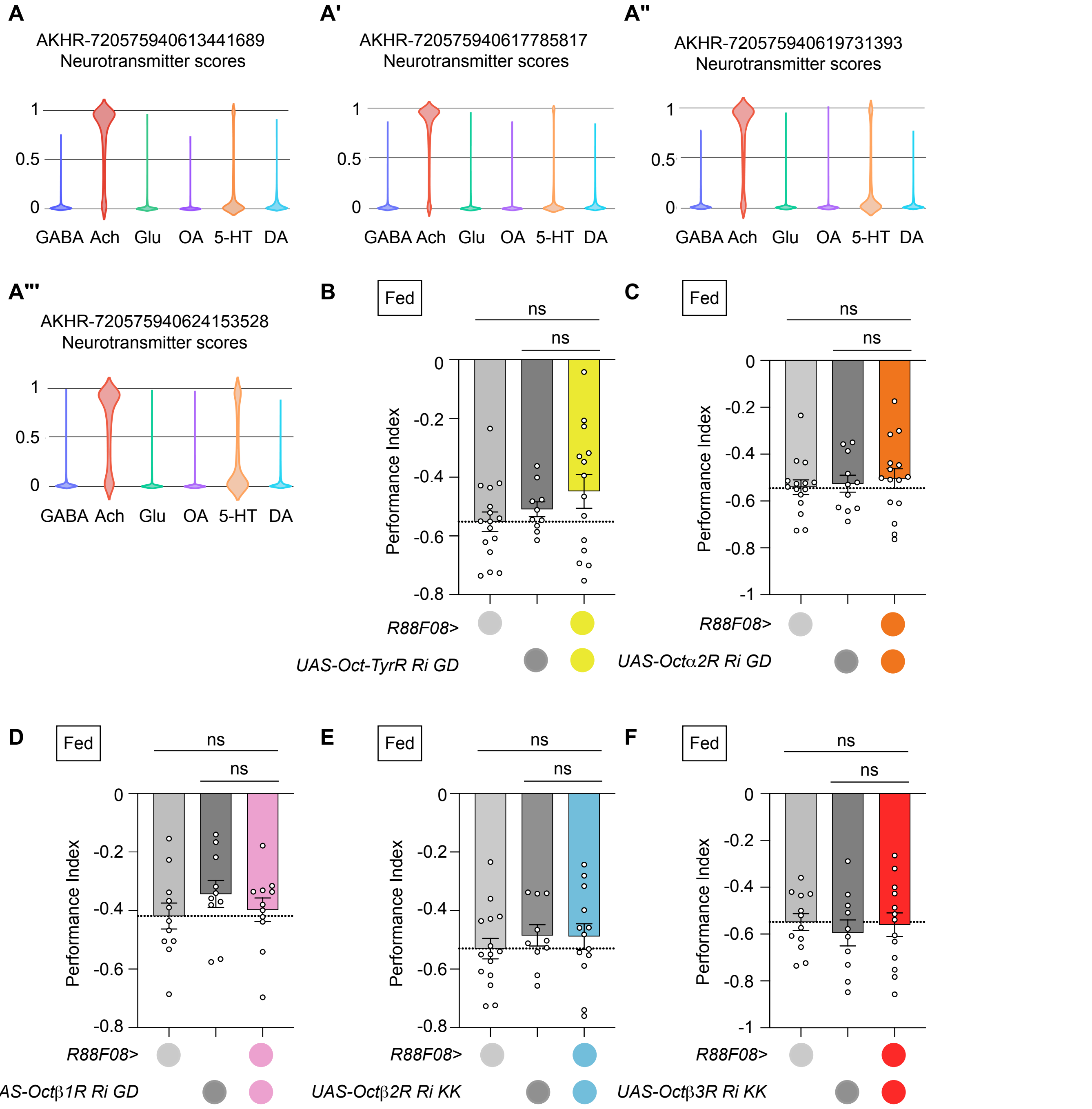

Figure S7, related to Figure 6.

Neurotransmitter prediction of Flywire AKHR neurons and test of other octopamine receptor requirement in SEZON01 neurons for aversive learning.

A-A'''. Neurotransmitter prediction scores obtained from Flywire (fly_connectivity tool), suggest that pre-synapses of all 4 AKHR neurons are most likely to be cholinergic, and secondly serotonergic. **A.** AKHR-720575940613441689. **A'.** AKHR-720575940617785817. **A''.** AKHR-720575940619731393. **A'''.** AKHR-720575940624153528. **B.** *Oct-TyrR* RNAi knockdown in SEZON01 neurons does not impact immediate aversive memory (*R88F08> Oct-TyrR Ri GD* flies), n≥10. **C.** *Octα2R* RNAi knockdown in SEZON01 neurons does not alter immediate aversive memory (*R88F08> Octα2R Ri GD*), n≥11. **D.** *Octβ1R* RNAi knockdown in SEZON01 neurons does not alter immediate aversive memory (*R88F08> Octβ1R Ri GD* flies), n≥10. **E.** *Octβ2R* RNAi knockdown in SEZON01 neurons does not alter immediate aversive memory (*R88F08> Octβ2R Ri KK* flies), n≥10. **F.** *Octβ3R* RNAi knockdown in SEZON01 neurons does not alter immediate aversive memory (*R88F08>Octβ3R Ri KK* flies), n≥10.

Data presented as mean ± SEM. Individual data points displayed as dots represent independent groups of approximately 200 flies.

Method S1: Original Code for analysis of $GRAB_{DA2m}$ imaging data, related to STAR Methods.

```
%%%%%%%%%%%%%%%%%%% 2P imaging data analysis%%%%%%%%%%%%%%%%%%
% Lucille Duquenoy 29/03/23

% input A with a frame number in column 1 (to be converted to time)
% shock detection in column 2
% odour info in column 3
% raw F of the ROI in column 4
% raw F of the background noise in column 5

frame_nb = A(:,1);
shock = A(:,2);
odour = A(:,3);
rawF_ROI = A(:,4);
rawF_background = A(:,5);

%% Calculate time from frame number and frame rate
time = frame_nb / 5.92; %frame rate = 5.92

%% Remove background from raw F of the ROI
rawF = rawF_ROI - rawF_background;


%% Shock response pre- & during odour presentation
%% Find time point at which the fly got shocked
i = 2;
time_shock_alone = [];
time_shock_with_odour = [];
while not(i == size(A,1));%while i different from size(A,1)=total length of
the expe
    if shock(i) >= 1.5*shock(i-1);
        if odour(i)<1000;
            time_shock_alone=[time_shock_alone,i-1];
             i=i+20; %add ~1.5s (1.5*5.92) to the counter so doesn't take in
account several times the same shock)
        else
            time_shock_with_odour = [time_shock_with_odour,i-1];
            i=i+10; %add ~1.5s (1.5*5.92) to the counter so doesn't take in
account several times the same shock)
        end
    else
        i=i+1;
    end
end


%% Baseline and gather shock response alone from rawF

x_shock = (-8:54)/5.92;
F_shock_alone = zeros(63,size(time_shock_alone(1,:),2));
baseline_pre_shock_average = 0;
for i=1:size(time_shock_alone(1,:),2);
    baseline_pre_shock = sum (rawF((time_shock_alone(i))-
8:(time_shock_alone(i)-3)),1)/6; %baseline for 1s (6frames) from 3 frames
before the start of the shock
    baseline_pre_shock_average = baseline_pre_shock_average +
baseline_pre_shock;
```

```
    F_shock_alone(:,i)= (rawF((time_shock_alone(i))-
8:(time_shock_alone(i))+54,1)- baseline_pre_shock)/ baseline_pre_shock;
end
baseline_pre_shock_average =
baseline_pre_shock_average/size(time_shock_alone(1,:),2);

F_shock_alone_average = zeros(63,1);
for i = 1:63;
    F_shock_alone_average(i,1) =
sum(F_shock_alone(i,1:size(F_shock_alone(1,:),2)))/size(F_shock_alone(1,:),2);
end

AUC_1_5s_shock_alone_average=trapz(F_shock_alone_average(7:17,:)); %from 1
frame before the stim to 1.5s after the stim
AUC_2s_shock_alone_average=trapz(F_shock_alone_average(7:23,:)); %from 1 frame
before the stim to 2s after the stim
AUC_3s_shock_alone_average=trapz(F_shock_alone_average(7:27,:)); %from 1 frame
before the stim to 3s after the stim
AUC_4s_shock_alone_average=trapz(F_shock_alone_average(7:32,:)); %from 1 frame
before the stim to 4s after the stim
AUC_5s_shock_alone_average=trapz(F_shock_alone_average(7:37,:)); %from 1 frame
before the stim to 5s after the stim
AUC_6s_shock_alone_average=trapz(F_shock_alone_average(7:43,:)); %from 1 frame
before the stim to 6s after the stim

figure (1)
plot(x_shock,F_shock_alone,'DisplayName','individual shock responses')
hold on
plot(x_shock,F_shock_alone_average,'LineWidth',2,'DisplayName','average')


%% Baseline and gather shock response when odours are ON from rawF

F_shock_with_odour=zeros(63,size(time_shock_with_odour(1,:),2));
baseline_pre_shock_with_odour_average = 0;
for i=1:size(time_shock_with_odour(1,:),2);
    baseline_pre_shock_with_odour = sum (rawF((time_shock_with_odour(i))-
8:(time_shock_with_odour(i)-3)),1)/6;
    baseline_pre_shock_with_odour_average =
baseline_pre_shock_with_odour_average + baseline_pre_shock_with_odour;
    F_shock_with_odour(:,i)= (rawF((time_shock_with_odour(i))-
8:(time_shock_with_odour(i))+54,1)- baseline_pre_shock_with_odour) /
baseline_pre_shock_with_odour;
end
baseline_pre_shock_with_odour_average =
baseline_pre_shock_with_odour_average/size(time_shock_with_odour(1,:),2);


F_shock_with_odour_average = zeros(63,1);
for i = 1:63;
    F_shock_with_odour_average(i,1) =
sum(F_shock_with_odour(i,1:size(F_shock_with_odour(1,:),2)))/size(F_shock_with
_odour(1,:),2);
end

AUC_1_5s_shock_with_odour_average=trapz(F_shock_with_odour_average(7:17,:));%f
rom 1 frame before the stim to 1.5s after the stim
AUC_2s_shock_with_odour_average=trapz(F_shock_with_odour_average(7:23,:));%fro
m 1 frame before the stim to 2s after the stim
```

```
AUC_3s_shock_with_odour_average=trapz(F_shock_with_odour_average(7:27,:));%fro
m 1 frame before the stim to 3s after the stim
AUC_4s_shock_with_odour_average=trapz(F_shock_with_odour_average(7:32,:));%fro
m 1 frame before the stim to 4s after the stim
AUC_5s_shock_with_odour_average=trapz(F_shock_with_odour_average(7:37,:));%fro
m 1 frame before the stim to 5s after the stim
AUC_6s_shock_with_odour_average=trapz(F_shock_with_odour_average(7:43,:));%fro
m 1 frame before the stim to 6s after the stim

figure (2)
plot(x_shock,F_shock_with_odour,'DisplayName','individual shock responses with
odours ON')
hold on
plot(x_shock,F_shock_with_odour_average,'LineWidth',2,'DisplayName','average')


%% Plot both averaged traces for shock pre- and during odour presentation

figure (3)
plot(x_shock,F_shock_alone_average,x_shock,F_shock_with_odour_average)
legend('pre-odour','during odour')


%% Odour response
%% Find time point at which the odour is ON
i = 2;
time_odour_on = []; %1st column will be odour alone and second will be odour +
shocks
while i<size(A,1);%while i different from size(A,1)=total length of the expe
    if odour(i)>=1000;
            time_odour_on=[time_odour_on,i];
            i=i+420; %add ~70s (70*5.92) to go to 2nd odour presentation)
    end
    i=i+1;
end

%% Baseline and gather odour response alone and with shocks from rawF

F_odour_alone=zeros(710,1); % odour presentation are 60s + 30s before and 30s
after = 120s*5.92 SO 178 before, 532 after)

baseline_pre_odour_alone = sum (rawF((time_odour_on(1))-63:(time_odour_on(1)-
3)),1)/61; % takes baseline for 10s from 1.5s before odour onset
F_odour_alone(:,1)=(rawF((time_odour_on(1))-178:(time_odour_on(1))+531,1)-
baseline_pre_odour_alone) / baseline_pre_odour_alone;

AUC_odour_alone=trapz(F_odour_alone(183:207,:));%from 6 frames after the stim
to 5s after (5*6=30 frames)



F_odour_with_shock=zeros(604,1); % odour presentation are 60s + 30s before and
12s after = 120s*5.92 SO 178 before, 425 after)

baseline_pre_odour_with_shock = sum (rawF((time_odour_on(2))-
63:(time_odour_on(2)-3)),1)/61; % takes baseline for 10s from 1.5s before
odour onset
F_odour_with_shock(:,1)=(rawF((time_odour_on(2))-
178:(time_odour_on(2))+425,1)- baseline_pre_odour_with_shock) /
baseline_pre_odour_with_shock;
```

```
AUC_odour_with_shock=trapz(F_odour_with_shock(183:207,:)); %from 6 frames
after the stim (since odour takes longer to reach the fly) to 5s after (5*6=30
frames)
%AUC_odour_with_shock=trapz(F_odour_with_shock(177:531,:))-
(AUC_shock_with_odour_average*size(time_shock_with_odour(1,:),2)); %from 1
frame before the stim to the end
%removing the average AUC of the shocks multiplied by the number of shocks


x_odour_alone=(-178:531)/5.92;
x_odour_with_shock=(-178:425)/5.92;
figure (4)
plot(x_odour_alone,F_odour_alone,x_odour_with_shock,F_odour_with_shock)
legend('1st odour presentation','2nd odour presentation')
hold on


zoom_x_odour_alone = (-12:60)/5.92;
zoom_x_odour_with_shock = (-12:60)/5.92;
zoom_F_odour_alone = F_odour_alone((165:237),:);
zoom_F_odour_with_shock = F_odour_with_shock((165:237),:);
figure (5)
plot(zoom_x_odour_alone,zoom_F_odour_alone,zoom_x_odour_with_shock,zoom_F_odou
r_with_shock)
legend('1st odour presentation','2nd odour presentation')
hold on


% to export
%multiple_AUCs=[AUC_1_5s_shock_alone_average,AUC_1_5s_shock_with_odour_average
];
AUCs_shock=[AUC_1_5s_shock_alone_average, AUC_1_5s_shock_with_odour_average;
    AUC_2s_shock_alone_average, AUC_2s_shock_with_odour_average;
    AUC_3s_shock_alone_average, AUC_3s_shock_with_odour_average;
    AUC_4s_shock_alone_average, AUC_4s_shock_with_odour_average;
    AUC_5s_shock_alone_average, AUC_5s_shock_with_odour_average;
    AUC_6s_shock_alone_average, AUC_6s_shock_with_odour_average];
AUCs_odour=[AUC_odour_alone,AUC_odour_with_shock];
Traces_Average_Shocks=horzcat(transpose(x_shock),F_shock_alone_average,F_shock
_with_odour_average);
Trace_Odour_only=horzcat(transpose(x_odour_alone),F_odour_alone);
Trace_Odour_with_shock=horzcat(transpose(x_odour_with_shock),F_odour_with_shoc
k);
Baselines_preshock_preodour=[baseline_pre_shock_average,baseline_pre_shock_wit
h_odour_average,baseline_pre_odour_alone,baseline_pre_odour_with_shock];
```
